# The furnace *and* the goat—A spatio-temporal model of the fuelwood requirement for iron metallurgy on Elba Island, 4th century BCE to 2nd century ce

**DOI:** 10.1371/journal.pone.0241133

**Published:** 2020-11-12

**Authors:** Fabian Becker, Nataša Djurdjevac Conrad, Raphael A. Eser, Luzie Helfmann, Brigitta Schütt, Christof Schütte, Johannes Zonker

**Affiliations:** 1 Department of Earth Sciences, Physical Geography, Institute of Geographical Sciences, Freie Universität Berlin, Berlin, Germany; 2 Mathematics for Life and Materials Sciences, Zuse Institute Berlin, Berlin, German; 3 Classical Archaeology (Winckelmann-Institute), Faculty of Humanities and Social Sciences, Humboldt-Universität zu Berlin, Berlin, Germany; 4 Department of Mathematics and Computer Science, Biocomputing Group, Mathematics Institute, Freie Universität Berlin, Berlin, Germany; University at Buffalo - The State University of New York, UNITED STATES

## Abstract

Scholars frequently cite fuel scarcity after deforestation as a reason for the abandonment of most of the Roman iron smelting sites on Elba Island (Tuscan Archipelago, Italy) in the 1st century bce. Whereas the archaeological record clearly indicates the decrease in smelting activities, evidence confirming the ‘deforestation narrative’ is ambiguous. Therefore, we employed a stochastic, spatio-temporal model of the wood required and consumed for iron smelting on Elba Island in order to assess the availability of fuelwood on the island. We used Monte Carlo simulations to cope with the limited knowledge available on the past conditions on Elba Island and the related uncertainties in the input parameters. The model includes both, wood required for the furnaces and to supply the workforce employed in smelting. Although subject to high uncertainties, the outcomes of our model clearly indicate that it is unlikely that all woodlands on the island were cleared in the 1st century bce. A lack of fuel seems only likely if a relatively ineffective production process is assumed. Therefore, we propose taking a closer look at other reasons for the abandonment of smelting sites, e.g. the occupation of new Roman provinces with important iron ore deposits; or a resource-saving strategy in *Italia*. Additionally, we propose to read the development of the ‘deforestation narrative’ originating from the 18th/19th century in its historical context.

## Introduction

In his seminal paper *The Furnace versus the Goat* published in 1983, Theodor A. Wertime put forward the notion that ‘pyrotechnological industries’—including metallurgy—were the main cause of deforestation in the Mediterranean region during antiquity [1]. Citing the case of Etruscan Populonia on the coast of Tuscany, Italy, Wertime follows two lines of arguments. First, he identifies economic strategies pursued by the Etruscans to cope with fuelwood scarcity. These includes the transport of raw ore to locations with available forest resources and the transition from energy-inefficient copper production to the more efficient iron smelting process [1, 2]. Second, Wertime estimates ‘energy costs’—i.e. the demand for woodlot—of iron smelting in Populonia based on the quantity of slag found there and a factor to estimate fuelwood requirements from the quantity of slag. The latter approach is common in studies on resource consumption in pre-industrial (charcoal-fuelled) iron metallurgy [2–13]; recent approaches focus on anthracological or palynological analysis [14–16].

**Fig 1:**
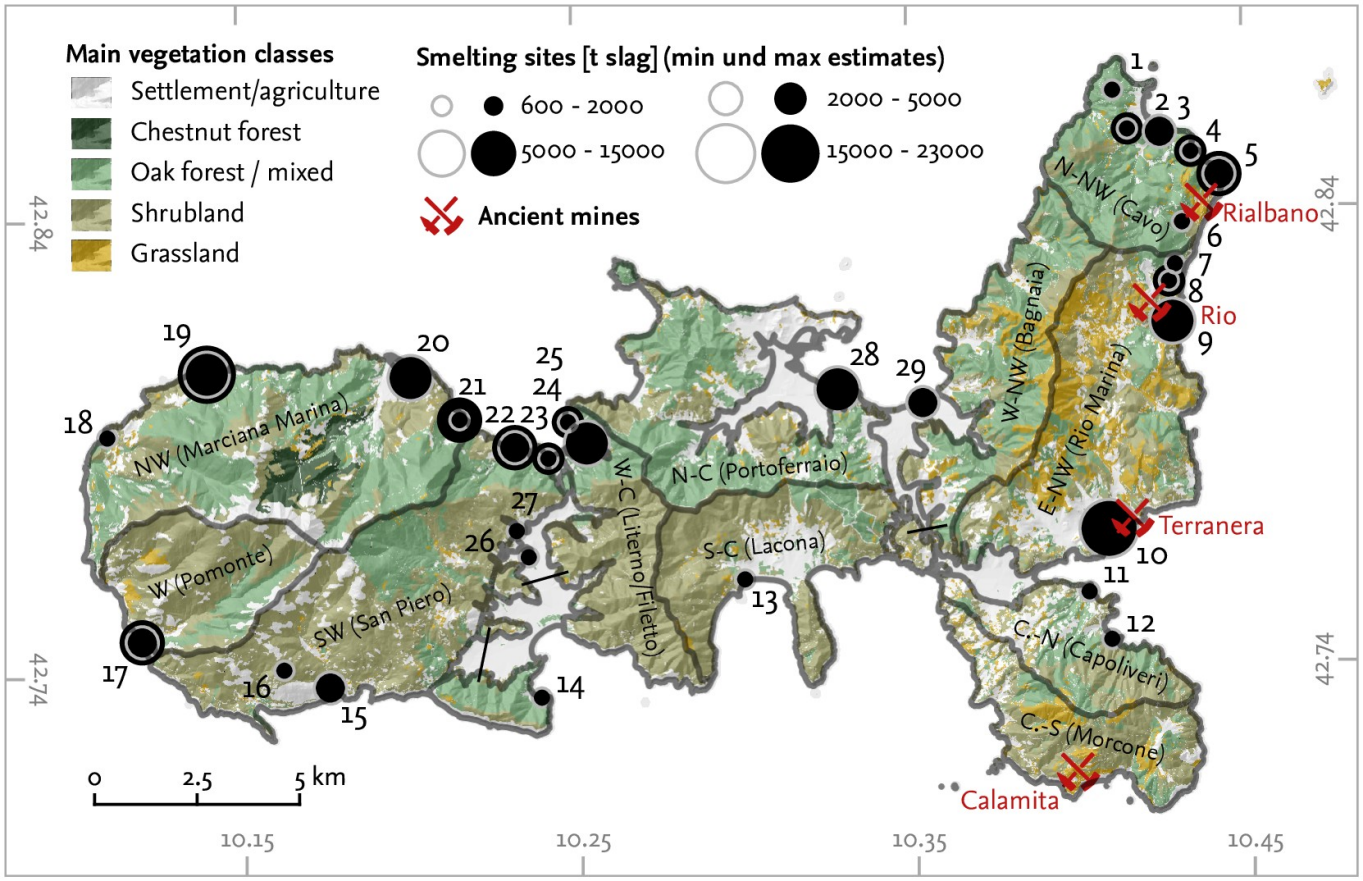
Ancient smelting sites, ancient mines, and main vegetation types on Elba Island. The size of the dots for each site is a function of the estimated quantity of slag disposed on each site during antiquity; black dots indicate upper estimates, whereas grey circles indicate lower estimates (see Table 2). The regions that were used for the model analysis in the *Results* section are shown (grey borders). IDs of the sites correspond to those given in Table 2. Data source (main vegetation classes): Regione Toscana—Data Base Topografico 1:10.000.

Romans and Etruscans exploited most of the iron that was processed in Populonia from the ore deposits on Elba Island (Fig 1). Historical texts and archaeological and archaeometallurgical records suggest that Elba Island played a key role in the supply of raw iron ore and iron bloom for the Etruscan and Roman economy (e.g. *Diod. Sic*. 5.13.1; *Str. 5.2.6*; *Verg. Aen*. 10.170; *Ps.-Aristot*. Mir. 93; [17–20]).

Ancient metallurgical centres are often regarded as hotspots of deforestation and subsequent fuel scarcity [21, 22], although some authors allege that deforestation is overestimated or of only local significance [14, 16, 23, 24]. Therefore, the reconstruction of past human–environment interactions plays a key role in current understanding of the development of iron metallurgy on Elba and Populonia. While the onset of iron smelting in Populonia dates to the 7th or 6th century bce [25, 26], the first smelting activities on Elba most likely date to the 4th century bce [27–30]. Scholars suggest several reasons for the time lag between the first smelting operations in Populonia and on Elba Island. First, an ‘industrial quarter’ for copper metallurgy in Populonia existed before the onset of iron production. Copper production on Elba Island, in contrast, took place only on a small scale between the 9th and 7th c. bce [31]. Thus, the proximity of the deposits on Elba Island to Populonia and the accessibility from the coast might have made the transport of the ore directly from the mines to Populonia an obvious choice. Moreover, political threats in the Tyrrhenian Sea (see [32, 33]) made Elba Island a precarious place at that time. Chiarantini et al. and Wiman [31, 34] raise the issue of a lack of fuel supply on Elba in the 6th century bce *(sic!)*. Also Veal [35] and Lang [36] believe that the reason for the transport of ore from Elba to Populonia in Etruscan or early Roman times was deforestation.

Iron production on Elba Island increased after Roman occupation in the mid -3rd century bce [33]. Ancient authors took notice of smelting activities on the island during Roman times (e.g. Diod. Sic. 5.13.1; see [17, 37]). Smelting sites dating to the Roman period were located all over the island, although iron mines on Elba are only located along the eastern coast [18] (see Fig 1). Scholars explain this pattern as being due to rational use of the fuelwood resources on the entire island [33, 38]. Occasional finds of forging slags in a contemporary hilltop settlement point to small scale refining on Elba Island for domestic use [39]; from Populonia, large forges are known [40]. Corretti [33] proposes that the location of smithing activities on the mainland also transferred some of the fuel requirement to the Apennine peninsula. Smithing—like smelting—required significant amounts of wood or charcoal. This fact, along with the location of the smelting sites on Elba, may therefore imply a strategy of rational fuel consumption. Mighall and Crew [41] proposed that bloomery smelters were interested in ensuring a constant fuel supply to maintain production.

In the mid -1st century bce, most of the smelting sites on Elba Island were abandoned [28, 33, 38, 42]. It is widely supposed that a lack of fuel caused the abandonment of smelting on the island [21, 23, 34, 35, 43–53]. Other authors mention the role of deforestation and the scarcity of resources as leading to the decline of metallurgy on Elba Island as well as in Populonia [54, 55]. These scholars mainly cite historical evidence to argue that a lack of fuel was the (main) driver of the decline of iron industries on Elba Island during antiquity. According to a report by the ancient Greek geographer Strabo dating to the late 1st century bce, the Romans did not produce iron directly on Elba, but they transported the raw ore to the mainland.

I myself saw these islands [i.e. Elba, Sardinia, and Corsica] when I went up to Poplonium, and also some mines out in the country that had failed. And I also saw the people who work the iron that is brought over from Aethalia [i.e. Elba]; for it cannot be brought into complete coalescence by heating in the furnaces on the island; and it is brought over immediately from the mines to the mainland.—*Strabo 5.2.6*; [56], *pp. 355–356*.

Scientific evidence for deforestation or a lack of fuel on Elba Island during the 1st century bce is sparse. Although data from palynological records indicate a decline in oak pollen [57], they do not suggest the disappearance of arboreal species [58–60]. Sedimentological data from alluvial deposits—surely only a limited direct indicator of deforestation—at least show that morphodynamics increased during the period of Roman iron smelting on Elba [61]. However, inconsistency in evidence for the ‘deforestation narrative’ of Elba Island indicate the need for additional data.

The entangled history of iron smelting and (alleged) deforestation ignited scientific debate in different contexts and fields. Archaeological examples of the scale of the impact of smelting on forests were reviewed by Pleiner and Iles [4, 6]. One example is the contribution of metallurgy to the Late Holocene Rainforest Crisis in Central Africa; here, the scale of iron smelting and contribution of climate change to forest transition is discussed using archaeological and environmental data [62–69]. A more recent example is the issue of West African metallurgy in colonial times. Research deals with the perspective of colonisers on a presumed deforestation by traditional smelters and the entanglement of deforestation, policy and economy [11, 70–74]. Similar examples are known from India [75]. Although in Britain and Germany in the 18th and 19th century, the issue of deforestation and iron smelting led to the development of forest policies; the existence of a “major timber scarcity” was later questioned [53, 76–83].

### Aims

Our main aim is to reconstruct the woodlot required for iron smelting on Elba Island in antiquity—guided by the question of whether the available wood resources were sufficient to supply the smelting sites with wood fuel. More specifically, we aim to:

1. setup a stochastic spatio-temporal model of energy and material flows and the land area required for fuelwood supply for iron smelting;
2. assess the variation of the model output related to the uncertainty of reconstructing past metallurgical processes and model parameters; and
3. develop a model including both the fuelwood consumption necessary to run the furnaces and the land area required to supply the labour force employed in smelting activities.

The focus of our analysis is the model setup and the uncertainty in the parameter specification. Developing Wertime’s dichotomy—*The Furnace* versus *the Goat*—we propose to take the contribution of both the furnace *and* the goat (i.e. food and fuel supplies for workers) into account when estimating the extent of ‘deforestation’ in a metallurgical hotspot. Browsing of leaves and young shoots by livestock—goat in particular—is regarded as causative for forest degradation and reduced regeneration in antiquity [49, 84, 85]. Ancient authors observed the destructive force of goats (*Theophr. Caus*. pl. 5.17.6; *Pl. Leg*. 1.639a; *Macrob. Sat*. 7.5.8–9). *Erica arborea*—a common wood species on Elba that was preferred by smelters [61, 86]—is more palatable than other macchia species [87, 88], due to the high tannin demand of goats.

### Pre-industrial iron metallurgy on Elba Island

Iron concentrations on Elba Island are synsedimentary to the middle–late Triassic Verrucano formation and were preconcentrated during metamorphism. Ores formed during the Late Miocene intrusion of plutons when iron was metasomatically-hydrothermally mineralised in saline fluids. Host rocks are mainly quartzites, phylittes and limestones; the deposits occur in stratiform bodies along north–south striking faults. Temperature and composition of the fluids varied during ore formation, resulting in a different composition of the deposits on Elba. The northeastern deposits are hematite-rich, whereas the deposits on the Calamita peninsula in the south-east are magnetite-rich (details on ore geology can be found in ref. [18, 89–91]). Iron ore deposits are generally limited to eastern Elba (Fig 1).

During antiquity, mainly the hematite-rich near-surface ores of the Rio and Rialbano deposits were exploited from the 6th century BCE. These ore bodies are made up of fine- to coarse lamellar or micaceous hematite that is often associated with pyrite and locally with Pb-Zn-Bi sulphides. Also hematite associated with pyrite and magnetite from the Terranera mines was potentially exploited in antiquity [18, 20]. The ore is generally of high quality suitable for extraction [4], Fe-contents of sampled ores range between 55% and 67% [10, 92]. The ore was smelted on Elba, but mostly on the Apennines Peninsula, e.g. in Populonia, Follonica-Rondelli, Genua and Pisa [93]. Three main periods of iron production on Elba can be identified, including the ancient period (6th c. BCE to 1st/2nd century BCE), a medieval period (11th to 14th century) and a modern period (mainly 18th century to 20th century) [48, 94]. Recorded descriptions of ancient smelting sites on Elba date back to a manuscript by A. Sarri form the mid-18th century [94]. Archaeological research on Elba dates back to the 19th century [42, 48, 95, 96]. During the excavation of the ancient iron slag heaps for re-smelting in the early 20th century, most traces of iron smelting on Elba were destroyed without sufficient archaeological documentation.

Iron ore was smelted on Elba in bloomery furnaces; furnace slags and fragments of furnace (walls) were found at almost all ancient smelting sites. Due to the sparse archaeological documentation of the archaeometallurgical record—most of the slag heaps were industrially excavated—, a detailed reconstruction of the furnace design and the operational conditions on Elba is challenging; no distinguishable “Elban” furnace type can be proposed. Reconstructions of the furnace type are based on in-situ finds of furnaces, but mainly on scattered archaeometallurgical finds. Hence, the detailed chronological development of furnaces during the ancient period can not be traced. However, some assumptions on the operation conditions and layout of bloomery furnaces on Elba Island can be made. First, from most ancient smelting sites, tapped slag has been recorded, pointing to slag tapping furnaces. Secondly, at some sites tuyères with small diameters were discovered indicating that furnaces with artificial air supply were in operation at least at these sites. Apart from these general points, a multitude of different furnace designs may have been in operation at the same period during antiquity [97]: At San Bennato (Fig 1, site 3) a circular furnace base of 0.7 m diameter (with a varying depth of 0.25–0.45 m) dug into the ground was excavated that dates to the 2nd/1st century BCE [27]. This furnace design is similar to the furnaces excavated at Follonica-Rondelli on the mainland. These furnaces presumably were semi-enterred low shaft furnaces driven by artificial air supply without slag tapping, and date to the late 6th/early 5th century BCE [98]. A form similar to this “Follonica”-furnace type has been recorded at the site of Gnacchera (Fig 1, site 13), but the top structure of this furnace indicates a little domed furnace [99]. Similar domed furnaces were also described by Wiman and Ekman at Populonia [100]. There, the circular oven structure contained blocks of local sand stone and burned clay, had a maximum height of c 0.4 m and was about 0.5 m in diameter. Evidence for slag tapping and for tuyères is lacking; the furnace has not yet been dated [100]. Based on archaeometallurgical finds collected during surveys and soundings, the so-called “Baratti”-type furnace is not only proposed for the smelting centre on the mainland, but also for San Giovanni (Fig 1, site 28) on Elba Island [101]. It is a free-standing low-shaft bloomery of c 1 m height and 0.3–0.4 m diameter with a single tuyère and a frontal hole for slag tapping [102]—a furnace type typical for the Roman era [103]. While quite similar in general layout, a different building material was used for the furnace in San Giovanni and in Baratti—whose reconstruction is also only based on scattered survey finds in Populonia. In San Giovanni much more clay material was used in the furnace structures whereas in Populonia local sand stone and clay were utilised [101] (this may be mainly due to local availability). The recent study of archive material of the Swedish researcher J. Nihlén who worked in 1957–61 on Elba revealed another furnace type. Nihlén recorded a big pit furnace of 1.5 m diameter and 1 m depth that was completely dug into the ground with a lateral air channel for artificial air supply in Sant’Andrea (Fig 1, site 19) as well as in Populonia [104]. This “Nihlén”-type furnace does not allow any slag tapping; only an “ancient” age can be proposed for this kind of furnace.

### Theoretical framework

The theoretical framework of our study is a socio-ecological perspective on past iron metallurgy [105] that developed from the concept of *societal metabolism* [106–108]. The application of the socio-ecological model to iron smelting on Elba Island is illustrative of current knowledge on the ‘deforestation narrative’ and reveals major research gaps. Moreover, the concept allows a formalisation of human–environment interactions on a material basis [109] and forms the basis of a numeric analysis of material and energy flows between a society and its physical environment (e.g. [110]). The model builds an appropriate framework for understanding dynamics in the land area required for human consumption.

**Fig 2:**
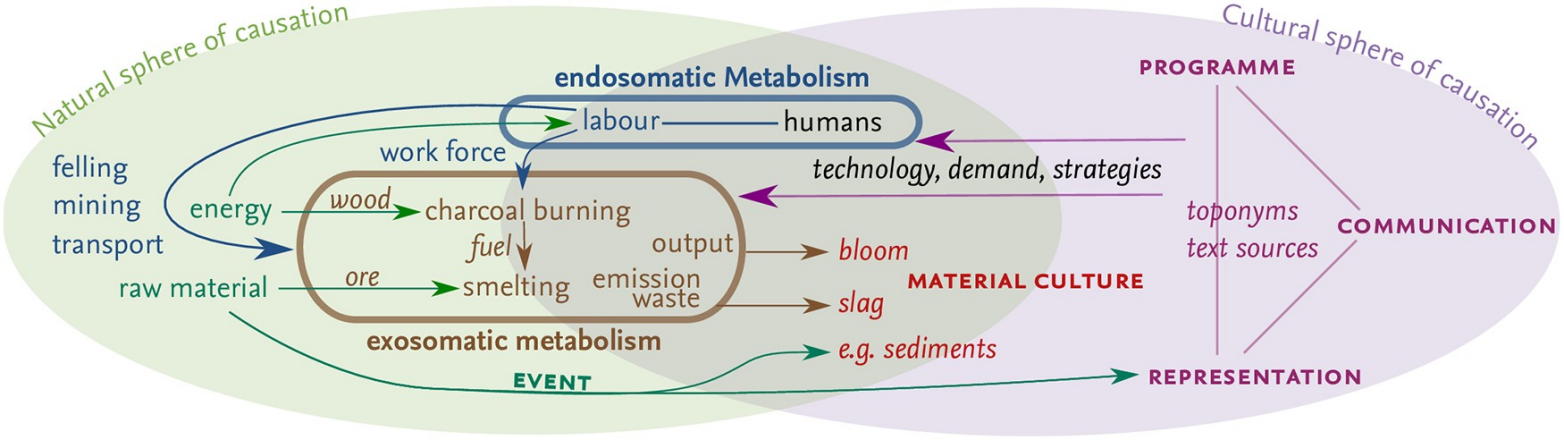
The socio-ecological model of iron metallurgy on Elba.

The socio-ecological model on iron metallurgy is based on the interactions between a natural and a cultural sphere of causation (Fig 2, [107]). The initial point of the model’s cultural sphere is the metallurgical *program*. This includes, for instance, the transition from copper to iron production, the development of the smelting technology, and the changing demand for iron. *Communication* between humans is crucial for the transfer of knowledge on iron production and the development of a strategy for the exploitation of resources. In addition, events related to metallurgical activities are communicated. *Representation* in the model is the record of recognised events or other aspects within the model. This includes e.g. written texts, epigraphs or toponyms. The basis of the model is—from an archaeological perspective—*material culture* (i.e. the remains of iron production, slag in particular). Material culture is indicative for past activities, but also allows conclusions on human agency to be drawn.

The natural sphere is the physical space in which humans are able to conduct the activities set in the program, including the availability and accessibility of (primary and secondary) resources. In the natural sphere, the effects of human interactions with nature are included in the model as *events* (*e.g*. flooding after clear-cutting, wood shortages or the emission of soot).

The interaction between nature and culture is described by the societal metabolism [106]. This concept from ecology includes the flow of energy and raw material, the conversion to a desired product, and the disposal of by-products (waste). The metabolism of iron metallurgy can be separated into an exosomatic (techno-)metabolism and an endosomatic (bio-)metabolism [109, 111]. The exosomatic metabolism includes not only the fluxes necessary for iron smelting (raw iron ore and charcoal in particular), but also the disposal of by-products (e.g. furnace remains, slag and charcoal fragments) and the emission of soot. The endosomatic metabolism comprises the energy necessary to provide nutrition for the humans involved in all operations of the metallurgical production processes, supervision, and supply. Material culture in the model is often a product of both, endo- and exosomatic metabolism.

Most models of the fuel requirement for iron smelting do not take the endosomatic metabolism into account and focus on the exosomatic metabolism. Models of energy requirements for e.g. life in cities, in contrast, focus on the endosomatic metabolism (e.g. [112, 113]). Recently, some authors have considered the entire societal metabolism [114–116]—these studies are nevertheless not conducted in areas with dominant metallurgical production. Saredo Parodi [10] and Cleere [3] analyse aspects of both endosomatic and exosomatic metabolism in the context of iron smelting—without, however, combining both. For Elba Island, the cultural sphere of causation, material remains and events are relatively well analysed, whereas (formalised) knowledge on the socio-ecological metabolism is limited.

## Model description

**Fig 3:**
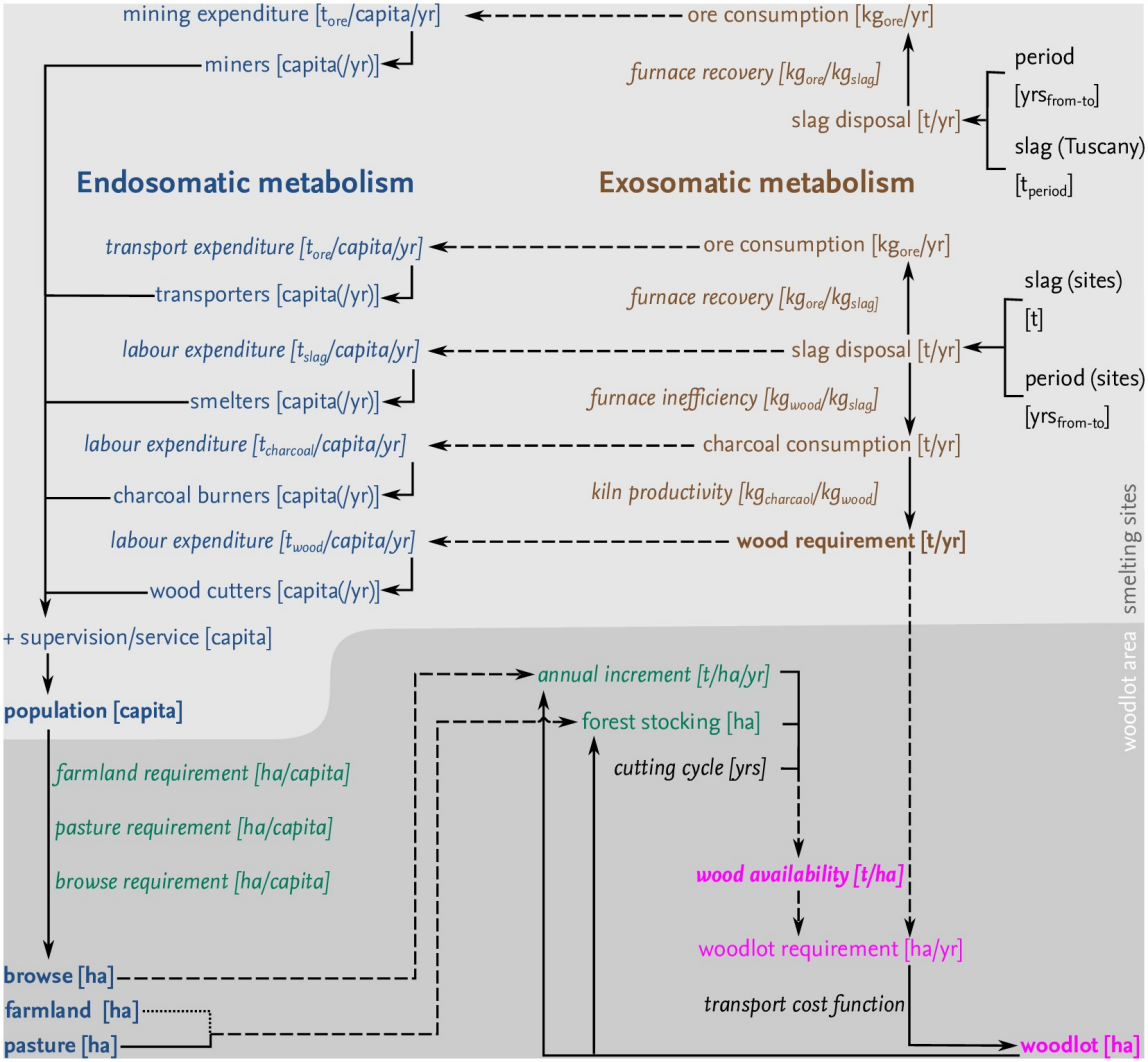
Sketch of the model.

Our modelling approach focuses on a reconstruction in space and time of the woodlot area required for the metabolism of iron smelting on Elba Island between 360 bce and 139 ce. The model is composed of three sub-models including (i) a sub-model of fuel demand for iron smelting (the exosomatic metabolism); (ii) a sub-model of the fuel demand and non-wood land use for supplying the workers employed in activities related to iron smelting (the endosomatic metabolism); and (iii) a sub-model of the requirements for workers employed in ore extraction on Elba Island (additional endosomatic requirements). Fuelwood availability is included in the model. The woodland area is reduced by cutting for fuelwood, clearing for food production and animal browsing (see Fig 3; Table 1). Annual regrowth is also modelled.

**Table 1:** Model parameters.

Parameter		Values	Distribution
Smelting	**Chronology**	site-specific, see Table 2	normal
	**Slag**	site-specific, see Table 2	normal
	Furnace inefficiency	2.0 to 8.5; mean 5.25±1.75 kg/kg (charcoal: slag)	lognormal
	Kiln productivity	Pit:.07 to.17; $\bar{x}=.11\pm .04$ kg/kg (charcoal: dry wood)	normal
		Mound:.135 to.28; $\bar{x}=.21\pm .07$ kg/kg (charcoal: dry wood)	normal
Mining	Ore extraction	0.97 to 3.67 Mt × 1.35 kg/kg (slag: ore)	uniform
	Mine chronology	varying intensity between 600 bce and 200 ce	*const.(ant)*
Labour	Mineworker	23 t/capita/yr (ore)	*const*.
	Charcoal burner	Pit: 3.5 t/batch, 12.5 person-days/batch	*const*.
		Mound: 45 t/batch, 25 person-days/batch	normal
	Woodcutter	13 ha/capita/yr (woodlot)	*const*.
	Smelter	2.5 t/capita/year (slag)	*const*.
	Transport	Animals: 187.5 t/capita/yr	*const*.
		Driver: 1 /5 animals	*const*.
		Seafarers: 300 t/capita/yr = 1 t/capita/day (ore)	*const*.
	Supervision	+30%	*const*.
	Service	+30%	*const*.
Food supply	Agricultural land	2 ha/capita	*const*.
	Pasture	transport animals: 0.8 to 1.1 ha/animal	*const*.
		Food supply: 0.72 ha/capita	
	Forest browse	0.69 ha/capita	*const*.
	Household fuel	0.5 to 2.0 t/capita/yr (charcoal)	*const*.
Wood supply	**Vegetation types**	spatially specific	–
	Rotation period	5 to 10 yrs	uniform
	**Increment**	age-dependent	*const*.
		Oak: 2.2 m^3^/ha/yr (10 yr mean)	*const*.
		*Macchia*: 3.6 m^3^/ha/yr (10 yr mean)	*const*.
		Chestnut: 5.7 m^3^/ha/yr (10 yr mean)	*const*.
	Specific gravity	0.69 t/m^3^ (dry wood)	*const*.
	Browsing	40–60% increment reduction	uniform

Parameters emphasised in **bold** are specific for Elba; other parameters are estimated for past conditions. For a detailed description of the parameters see Supporting information (unspecific parameters) and the main text (specific parameters).

### Uncertainty analysis

When modelling in the archaeological context, it is of special interest to include the uncertainty of the input parameters, because data is mostly sparse and validation against known data is often impossible. Historical data, for instance, only allows a rough qualitative assessment of the model outcome. There are not exact data on the operational conditions and inefficiencies of ancient smelting; only experimental data is available, which is based on the reconstruction of ancient techniques. Therefore, it is necessary to include uncertainty of the parameter estimates in the model. We use a data-centred approach to quantify uncertainty, thus focusing on the input data as a source of uncertainty. Other sources of uncertainty such as the choice of model parameters or model mechanisms (see [117, 118]) are not part of our stochastic model; we evaluate them qualitatively in the *Discussion* section. Uncertainty in our case is not to be confused with variability. Whereas uncertainty is related to a lack of current knowledge about production conditions, it is reasonably clear that iron production in antiquity was (highly) variable. This variability may be related to random differences in a single batch, but may also be due to inter-subject differences, and technological development. Our model does not cover this variability explicitly.

We undertook Monte-Carlo simulations, i.e. repeated simulations for different sets of parameters that are drawn randomly from a given distribution (see e.g. [119–123] for probabilistic models of uncertainty in archaeological dating). The distributions for each parameter are estimated from the archaeological findings (Table 1, last column). Since we have no detailed information on the *true* values valid for ancient Elba, we followed Laplace’s *Principle of insufficient reason*. A Gaussian distribution of the parameter values centred around the mean between the estimated possible extreme values, with variances according to the interval length, is assumed to fit to Laplace’s principle in our case.

Given the simulation results, we then evaluated the likelihood (i.e. the percentage of simulation runs) that an insufficient amount of wood was available for smelting on Elba based on the scheme proposed for the IPCC report [124].

### Exosomatic requirements

The basic units of the model are the ancient smelting sites on Elba (Fig 1). We used the estimated slag disposal and the chronology of each site (Table 2) to calculate the site-specific annual slag disposal during the period of interest. Based on a furnace inefficiency factor obtained from smelting experiments, we calculated the amount of charcoal necessary for the smelting activities on each site in each year. Using a productivity factor of charcoal kilns taken from a literature review, we obtained the fuelwood demand of a site in each year. Kiln productivity is the quantity of wood necessary to produce one unit of charcoal.

**Table 2:** Chronology and slag disposal at ancient smelting sites on Elba Island.

Site		Chronology [-BCE/CE]				Slag [t]			
ID	Name	*t*_*α*_	*t*_*β*_	*t*_*γ*_	*t*_*δ*_	min	max	Meth.	Ref.
1	Martella ant.	-360	-280	-211	-180	400	1400	*a;c*	[104]
2	Ombria	-300	-232	-93	-25	1000	5000	*e,g*	[32]
3	San Bennato	-300	-180	138	139	100	5000	*b; e,g*	[27, 32]
4	Fornacelle	-140	-10	73	73	1000	5000	*e,g*	[32, 38]
5	Capo Pero	-300	-201	-47	100	4400	13500	*b*	[32, 42]
6	Fegatella	-300	-300	-25	-25	600	1900	*a; b*	[48, 95, 104]
7	Valle del Giove	-300	-140	-25	-10	600	1200	*a*	[104]
8	Vigneria	-300	-226	-75	-1	1000	5000	*e,g*	[32]
9	Rio Marina / Spiazzi	-225	-145	1	50	100	15000	*b; e*	[32, 38, 42, 95]
10	Barbarossa	-260	-210	-120	-50	20500	20500	*d*	[104]
11	Naregno	-260	-248	-222	-210	1200	1200	*d*	[104]
12	Straccoligno	-260	-210	-200	-120	1000	1000	*d*	[104]
13	Lacona	-140	-140	-10	-10	600	1200	*a*	[104]
14	Galenzana	-130	-123	-107	-100	600	1200	*e*	[104]
15	Seccheto	-280	-217	-88	-25	2600	4300	*f*	n/a
16	Sughera	-280	-217	-88	-25	100	1000	*c*	n/a
17	Pomonte	-130	-100	-100	-50	4800	14700	*f,c; b,c*	[38, 42, 129]
18	Patresi	-260	-140	-120	-10	1000	2000	*c*	n/a
19	Sant’Andrea	-200	-120	-101	-10	10000	23000	*d; b*	[104]
20	Marciana Marina	-290	-240	-60	-10	9000	10500	*b*	[32]
21	Bagno	-220	-175	-85	-40	2000	10000	*g*	[32]
22	Paolina	-200	-135	-60	60	5000	15000	*g*	[32]
23	Gnacchera	-300	-232	-93	-25	1000	5000	*g*	[32]
24	Guardiola	-204	-140	-120	-10	1000	5000	*g*	[32]
25	Campo all’Aia	-130	-100	-60	-20	10800	10800	*d*	[104]
26	La Pila	-140	-108	-42	-10	300	600	*b*	[38]
27	Forcioni	-140	-50	-10	100	400	1800	*c*	[38, 42]
28	San Giovanni	-200	-120	15	100	10500	14500	*d; b*	[42, 94, 104]
29	Magazzini	-300	-165	-60	60	200	4000	*b*	[94]

Uncertainties as outlined in the main text and related references are given. Details on the dating material used to define the dating range can be found in the Supporting information (references: [27, 32, 38, 42, 94, 95, 104, 153–156]). Age estimation: *t*_*α*_ = lower bound, *t*_*β*_ = lower start, *t*_*γ*_ = lower end, *t*_*δ*_ = upper bound. Methods to estimate quantity of slags: a Characterisation by re-smelting concessionaire as economically viable or as ‘significant but low quantity’, i.e. ca. 600–1200 t; b area extent /volume given in literature (slag not removed); c own measurements of slag accumulation; d data from re-smelting concessionaire; e (own) classification as small site; f estimation of locals, area with small density of slags; g published estimation.

#### Smelting sites

We compiled a list of ancient smelting sites on Elba (*n* = 29) containing all finds of material dating to the Etrusco–Roman smelting period (*n* = 96), *viz*. ceramics, coins, ^14^C-ages, and stratigraphic position. The date density per site ranges from a single isolated find (e.g the Naregno site [38, 42]) to well dated excavated sites (the San Bennato site [27, 42]). If dating material clearly predated the general chronology of smelting on Elba (4th/3rd century bce to 2st century ce), the material was excluded from our data set; some sites were not exclusively used for smelting.

**Fig 4:**
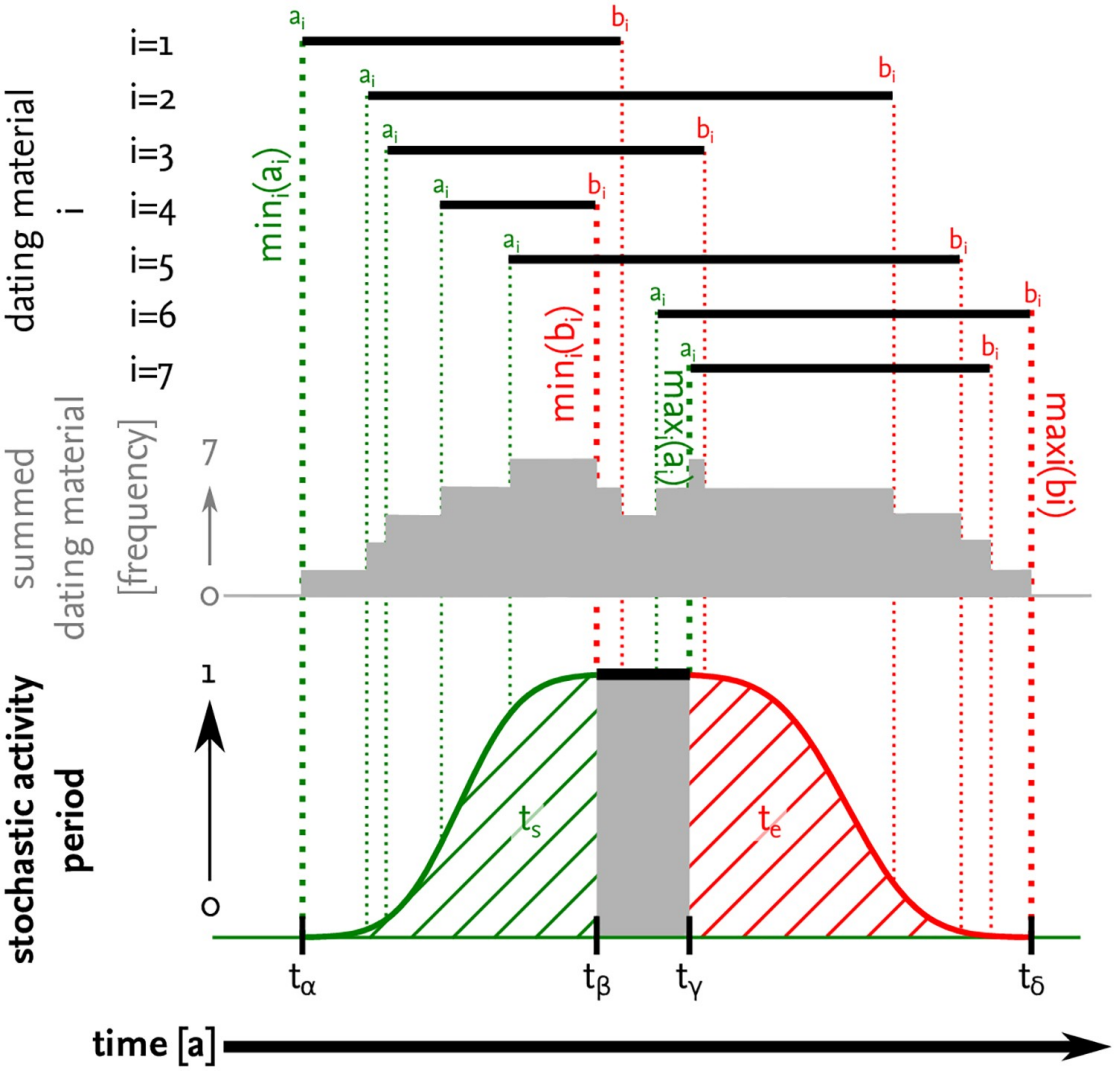
Estimation of the activity period for an example smelting site.

The absolute date of the deposition is unknown for most of the dating material. For instance, the production of Dressel 1A amphorae conventionally dates between the mid-2nd and mid-1st century BCE (ca. 140–50 bce) [125, 126]; *Terra sigillata italica* was mainly produced between ca. 60 bce and 60 ce [127]. The dating material thus only indicates a possible time span for the *true* moment of deposition and, so, the operational phase of a site. In order to deal with this issue, we used a probabilistic approach to incorporate the uncertainty of dating into our model input (Fig 4).

We assume that the activity for a single site is monophasic. For each site and each simulation run, we draw the initial point *t*_*s*_ and the end point *t*_*e*_ of the activity time span from normal distributions. From the data of each site we can estimate possible time frames Since we want the start and end date to lie within the chosen time frames, we redraw the random variable whenever values that lie outside are drawn. *t*_*α*_ ≤ *t*_*s*_ ≤ *t*_*β*_ of starting dates and *t*_*γ*_ ≤ *t*_*e*_ ≤ *t*_*δ*_ of end dates with *t*_*γ*_ > *t*_*β*_. We choose the mean *μ* of each time frame as the centre of the distributions and $\frac{1}{6}$ of the interval length as the standard deviation *σ* for the distributions resulting in

$$\begin{array}{cc} & t_{s}∼N(\frac{t_{\beta }+t_{\alpha }}{2},{\left(\frac{t_{\beta }-t_{\alpha }}{6}\right)}^{2})\\ & t_{e}∼N(\frac{t_{\delta }+t_{\gamma }}{2},{\left(\frac{t_{\gamma }-t_{\delta }}{6}\right)}^{2}) \end{array}$$

We then set the activity period for a site to be the interval [${\tilde{t}}_{s}$, ${\tilde{t}}_{e}$], where ${\tilde{t}}_{s}$, ${\tilde{t}}_{e}$ are the drawn random numbers rounded to the next integer. The times for the boundary values of the intervals are estimated from the archaeological findings of the site (see Supporting information). Each finding *i* is dated with a time interval [*a*_*i*_, *b*_*i*_]. We choose *t*_*α*_ = min_*i*_{*a*_*i*_}, *t*_*δ*_ = max_*i*_{*b*_*i*_} and the time interval [*t*_*β*_, *t*_*γ*_] as the smallest interval such that it intersects with all [*a*_*i*_, *b*_*i*_] in at least one time point. For our data also *t*_*β*_ = min_*i*_{*b*_*i*_} and *t*_*γ*_ = max_*i*_{*a*_*i*_} holds for all sites with multiple findings. In the case of only one finding at a site we choose $t_{\beta }=a_{1}+\frac{b_{1}-a_{1}}{4}$ and $t_{\gamma }=b_{1}-\frac{b_{1}-a_{1}}{4}$ in all simulations.

#### Amount of slag

Ancient slag from Elba contains high amounts of iron, which could be reduced in modern blast furnaces. Therefore, old slag heaps were mined and slags re-smelted in the first half of the 20th century [128]. The amount of slag removed from ancient heaps was well documented by the slag mining concessionaire I. Sapere (J. Nihlén’s records [104]; *n* = 6). Figures range from 1,000 t (Straccoligno site) to 20,500 t (Barbarossa site). For some slag accumulations excavated by the concessionaire, no exact figures are documented. The concessionaire described the slag heaps as ‘of small quantity which is still to be excavated’ [104]. This description is used in other cases for slag accumulations of between 600 and 1,200 t. As Nihlén’s data is based on figures from I. Sapere, who worked on Elba between 1938 and 1943 [128], some slag heaps were removed prior to documentation; these numbers are not included in our data set. We used the documented areal extent and depth of the slag heaps/accumulations to estimate the quantity of slag deposited at sites where no figures from the concessionaire are available ([32, 38, 42, 94, 104, 129], and own data). To calculate the slag weight *W*_*i*_ of a site from the areal extent and the average height when accessible, we used the following approach [130, 131]:

$$\begin{array}{c} W_{i}=\left(\left(h\times \delta \right)\times a\times b\right)\times Q\times \rho \end{array}$$

where *a*×*b* is the area of the slag accumulation, *h* is the height/depth of the accumulation, *δ* = (.41,.75) is a topographical correction factor for the irregularly shaped underground and surface of the accumulation, *Q* = (.24,.4,.5) is the ratio of slag to debris in an accumulation, and *ρ* = (2.0, 2.2) t/m^3^ is the density of slag. Values of constants were taken from the literature [131–133]. The depth correction obtained from Humphris et al. [132] is calculated as the average of the ratio between the topographically obtained volume of the accumulation and the volume obtained by resistivity measurements or excavations. To estimate the slag amounts at all other sites for which no areal extent of the slag accumulation is available, we used the data estimated by Zecchini [32].

The uncertainty assessment of the slag amount is based on minimum and maximum values of the calculations applying the equation for *W*_*i*_ and the range of published estimations. For sites where the extent of the slag accumulations and data from the slag mining concessionaire are available, we used the numbers of both data sets. Following Laplace’s *principle of insufficient reason*, we assume a normal distribution of the *true* slag amount between the extreme values estimated for each site *i*:

$$\begin{array}{c} \hat{W_{i}}∼N(\frac{W_{max}+W_{min}}{2},{\left(\frac{W_{max}-W_{min}}{6}\right)}^{2}) \end{array}$$

#### Furnace inefficiency

For our model, we define furnace inefficiency as the ratio of charcoal charge to the amount of slag removed from the furnace after smelting. Slag is the most common archaeometallurgical remain of smelting and is well documented in the archaeological record. The calculation of the furnace inefficiency is based on a compilation of in total 62 smelting experiments by F. Nikulka [134]. The data base covers experiments with different ore and furnace types from different regions in Europe and explicitly includes data on charcoal consumption (for both pre-heating and charcoal-ore charge and the amount of slag accumulated during a batch). In our model, we took only those experiments into account that had realistic mass balances (see Supporting information) or that were successful (i.e. experiments that resulted in the formation of a bloom). We used only the most efficient experiments if several experiments were conducted by the same operators and in similar conditions.

For the model, we only used the highest and lowest value in the data set (Table 1). The *true* furnace inefficiency used in a model run is randomly drawn from that value range. The probability that a value is sampled follows a right-skewed normal distribution. This kind of distribution is used because we assume that the real inefficiencies were lower than the experimental ones due to the long experience of the ancient smelters. Basing the approach only on high and low values from a set of smelting experiments pays tribute to the fact that the design of Etruscan/Roman furnaces on Elba is unclear (see above) and avoids calculating descriptive statistics from heterogeneous experiments with diverse settings.

Furnace design contributed to the efficiency of smelting; smelting in shaft furnaces might have consumed less charcoal than smelting in a pit furnace [4, 134]. Also the furnace diameter [135] as well as slag tapping [136] and air supply [135, 137] might have influenced efficiency. However, skill of the smelters [136], ore mineralogy [4, 136], charcoal quality and the iron economy [137] are also important determinants of the efficiency. A quantification of the contribution of these factors to the furnace inefficiency parameter is not possible at the moment.

#### Kiln productivity

We estimated kiln productivity on the basis of ethnographic records and charcoal burning experiments; kiln productivity is here defined as the proportion of charcoal gained from smelting one unit of dry wood. We used average values of two types of kilns, viz. pit kilns and earth mound kilns. Both types are testified in ancient texts. For pit kilns, few ethnographic records are available. We therefore rely on the data obtained by Horn [138] (up to .17 kg/kg—data from [139] are similar). We took into consideration that the production of high quality charcoal for iron smelting might have been less productive [140, 141], thus allowing the kiln productivity to be as low as .07 kg/kg. For earth mound kilns, values summarised for archaeological conditions range between .135 kg/kg and .28 kg/kg [5, 142]. With modern (traditional and non-traditional) charcoal burning technologies, productivity is at the higher end of the range or exceeds it [139, 143–146] (up to .32 kg/kg).

To account for the uncertainty in the estimation of kiln productivity, we randomly sampled a *true* value from the range of values as stated above. The probability that a value is sampled follows a normal distribution; the mean of the normal distribution is here set to half the range of all possible values and the standard deviation is set to 16 of the range [113]. We prefer the normal distribution for the productivity parameter to the continuous uniform distribution as efficiencies from ethnographic records (or even data from archaeological experiments) might be much higher than ancient productivities due to technological development. Low values (from experiments) in contrast might also misleading, as charcoal burners on Elba Island were most likely experienced specialists.

### Endosomatic requirements

The labour requirement is calculated separately for the main steps of the metallurgical processes, including mining, furnace operation, charcoal burning and wood felling. In addition, labour investment is necessary to transport material from the site of extraction (mines, forests) to the site of production (kilns, smelting sites) and for security. In additional, more persons lived on the island that e.g. provided food for the workers. Estimates of the work force are based on the quantities estimated for the different production steps (see Fig 3). The ore charge is calculated as the ratio of ore to slag for each furnace; the number of furnaces is calculated from the number of workers typically necessary to handle one furnace with a defined charge. The estimate of the work force for mining is based on estimated quantities of slag found around the Gulf of Follonica (including Populonia–Baratti and Poggio Butelli, which were the most important known production centres on the mainland, and Elba). For each labour group, we applied a specific factor of the annual *per capita* productivity (Table 1). From the number of workers required in each year and the number of additional persons living on the island (e.g. for security, services and supply), we obtained the total amount of fuelwood necessary for heating and cookin; further details on the calculation can be found in the Supporting information. These requirements contribute to the total fuelwood demand for the entire metabolism of iron smelting.

### Woodlot area and wood availability

Woodlot requirements are calculated as a function of the demand at each smelting site in each year and the available wood on the land around it. The pattern of available wood is initially based on the current distribution of the main vegetation classes on Elba (oak forest, Mediterranean *macchia* shrubland, and sweet chestnut forest, see Fig 1). We assume that the current vegetation cover roughly corresponds to the pattern at the onset of the smelting period, as the potential natural vegetation is also deciduous and evergreen oak forest [147]. Elba is located in the *Central and Northern Tyrrhenian Ecoregional Section* which is locally dominated by Mediterranean and Sub-Mediterranean evergreen shrublands of the Italian peninsula [148]. Dominant species are e.g. *Quercus ilex*, *Phillyrea latifolia*, *Arbutus unedo*, *Erica arborea*, and *Pistacia lentiscus*. Palynological data suggest that during the Etrusco–Roman period, as today, vegetation was dominated by *Ericaceae* shrubland and oak forests [57]. Since the 1950s forests have regenerated following a typical succession [149], mainly because the primary sector lost its importance on the island and tourism became the main source of income. For our model, we assume that today’s grasslands were covered with *macchia* shrubland at the beginning of the smelting period. We fitted a sigmoid function to average values of the (age-specific) annual increment in each of the vegetation classes to calculate the standing volume (at a tree age of 42 yrs) [150, 151]; we applied a constant specific gravity of wood to obtain dry mass from the standing volume. The selection of the felling area is based on the Pandolf least-cost function for positive and the Pandolf-Santee equation for negative slopes (walking speed is calculated using Tobler’s hiking function) [152]; areas with the lowest transport cost are preferably felled. The available woodland area was reduced by the area necessary for agricultural and horticultural production (field cultivation and animal grazing). We modelled the regrowth of the woodland for each year after harvest using the sigmoid function. The minimum age for harvesting is set to 5 yrs, which fits with ages for coppicing reported in ancient literature; Plin. HN 17.147–159 reports rotation periods for *Castanea sativa* (7 years) and *Quercus* (10 years); Columella [23] reports similar cycles (*Castanea*, 5 years; *Quercus*, 7 years). We define the moment of a lack of fuelwood as the year when the fuelwood requirement exceeds the available wood biomass on Elba.

### Simulation details

We constructed a grid from the vegetation increment data with a 10 m×10 m-resolution. All cells with woodland vegetation have a variable for the tree type and age. From these data we can determine the estimated available woodland through the previously described sigmoid function approach and establish whether the rotation period for felling has finished or forest browse is possible. For each site a distance measure is constructed that determines the distance to nearby cells in terms of transport costs using the average slope and distance of the direct path as input.

The first step of each simulation involved drawing the parameters as described in the previous section and determining from them the number of time steps, the activity periods and woodlot requirements of each site according to the model description. The time step size was chosen as one year and we assume that the woodlot requirement of a site is the same for all time steps.

For each time step of one simulation, all active sites were determined and according to their woodlot requirements the nearest (with respect to the previously constructed site-specific measure) cells with tree ages older than the respective rotation period were used for wood collection. For all cells that were chosen for wood collection or forest browse, the tree age variable was set to zero. After saving the current state the regrowth step was initiated and for each cell the tree age was increased by one year (to a maximum of 42 yrs). The simulation stops either because of complete deforestation (i.e. when there is no cell with a tree age older than the rotation period, while the required woodlot has not yet been met) or when the end of all site activity periods.

## Results

### Overall model outcomes

We undertook 11,479 simulations with parameters chosen as described in the model description section. From these we can estimate the metallurgical activities and the availability and distribution of woodland on Elba Island. The estimated amount of slag disposed of on Elba Island during antiquity ranges between 122,770 t and 173,500 t with an average of 146,170 t±6,464 t (Mdn±MAD). The minimum availability of woodland area (trees >5 yrs) in the simulation runs ranges between 0% and 62.9% of the undisturbed woodland area (41.1% ± 11.0pp); the minimum availability of mature woodland area (trees >42 yrs) ranges between 0% and 58.9% (23.4%±17.5pp). Between 4×10^−4^% and 30.4% (5.5%±2.2pp) of the original woodland area is estimated to be cleared within one year of operation.

### Likelihoods

**Fig 5:**
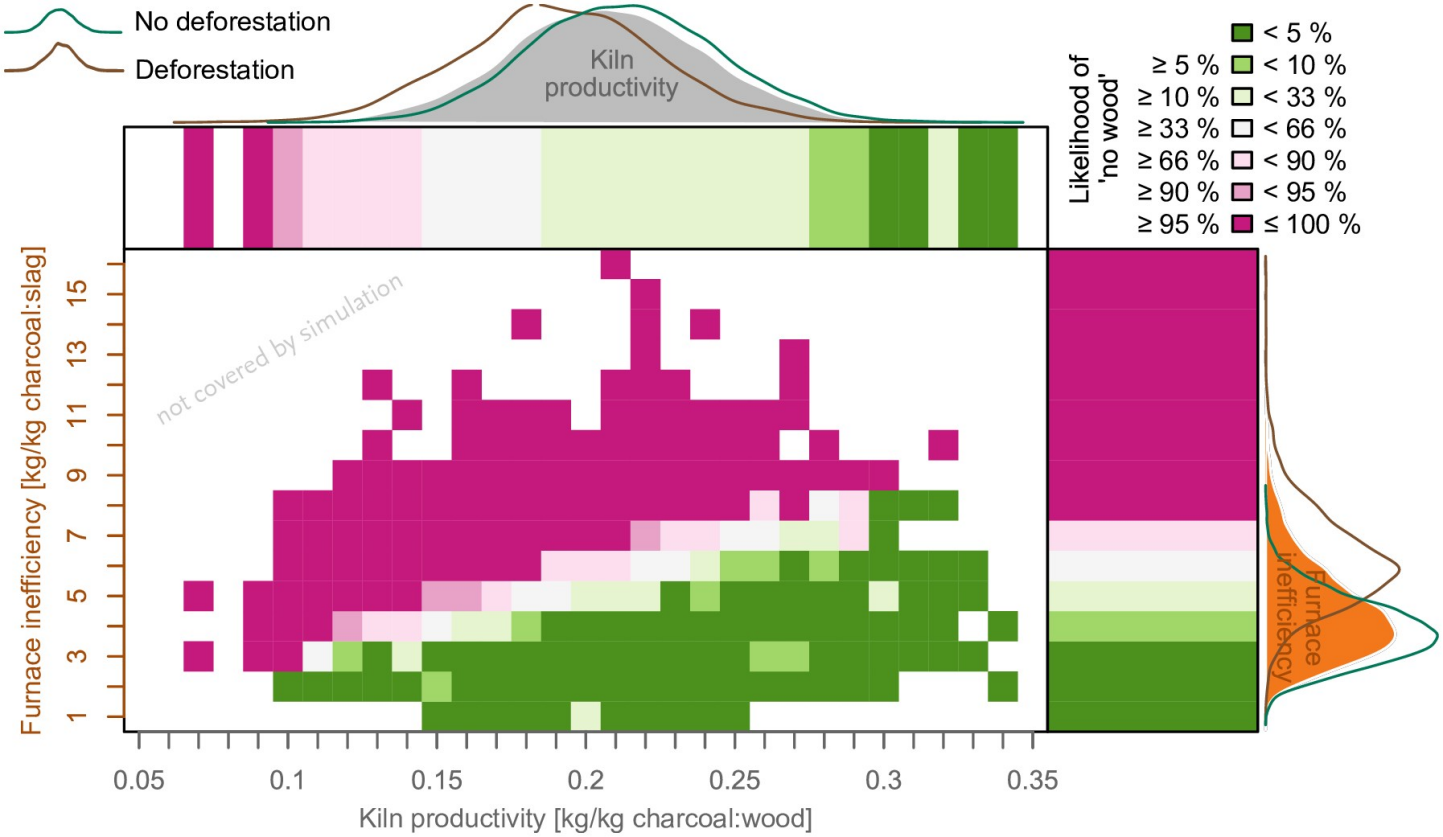
The likelihood that were was insufficient fuel for smelting on Elba between the 4th century BCE and the 2nd century CE in relationship to the main technical input parameters of the woodlot model. The density plots show probability distribution of the kiln and the furnace parameters for all simulations (grey), for simulations where insufficient wood is available for smelting (brown) and where no lack of wood occurred (green). The bars below the density plots show the likelihood of a lack of fuel for the furnace or the kiln parameters; the raster for a combination of both parameters.

The likelihood (i.e. here the percentage of simulation runs) that insufficient wood was available for charcoal production on Elba is 14.1% (exosomatic metabolism; evaluated as *unlikely*). When taking both the exo- and endosomatic metabolism into account, the likelihood that an insufficient amount of wood was available is 27.7% (evaluated as *unlikely*). The uncertainty of each parameter contributes to a different extent to the range of the model output (Fig 5).

The uncertainty in the estimation of site chronologies and total slag does not contribute importantly to the variability of the model output. The uncertainty in kiln productivity and furnace inefficiency has a major impact on the model outcome. The furnace inefficiency in simulations resulting in a complete disappearance of trees is 1.6 times higher than the inefficiency in simulations where not all woodland area is cleared. The relative difference is smaller when comparing kiln productivity (factor 1.12) or total amount of slag (factor 1.009) of the two classes. The relative MAD of values of the input parameters varies likewise (see Tables 1 and 3).

**Table 3:** Uncertainty of the parameter and model outcomes and likelihoods of the availability of woodland (trees older than 5 yrs) for given parameter values.

Parameter	%RMAD	%RD
Slag	6.1	1.1
*Furnace inefficiency*	35.4	3.7
Charcoal	37.4	4.7
*Kiln productivity*	16.8	1.3
Wood	40.1	5.0
%Trees (>40 yrs)	74.7	2.4

%RMAD = relative median absolute deviation (percent MAD divided by median), %RD = relative difference (percent range divided by median).

Taking the simulations that include the supply of the work force, it is *extremely unlikely* that all woodland on Elba was cleared if furnaces were (on average) less inefficient than 3.7 kg/kg, at least *unlikely* if inefficiency was <4.9 kg/kg and *likely* if the inefficiency was >6.0 kg/kg (see Fig 5 and Table 3). If the kiln productivity was greater than 0.29 kg/kg, 0.27 kg/kg, or 0.19 kg/kg it is *extremely unlikely*, *very unlikely*, or *unlikely*, respectively, that an insufficient amount of wood for smelting was available on Elba. It is *likely* that all woodland was cleared if the kiln productivity was <0.14 kg/kg. Lower efficiency and productivity are required when only exosomatic requirements are taken into account (Table 4). In Fig 5 a heat map highlights combinations of parameters for furnace inefficiency and kiln productivity and the respective likelihoods that no woodland area with sufficient wood for smelting on Elba was available in antiquity.

**Table 4:** Likelihoods of the availability of woodland (trees >5 yrs) for given parameter values. See also Fig 5.

Likelihood		Furnace inefficiency [kg/kg]		Kiln productivity [kg/kg]	
Term	Outcome	labour	no labour	labour	no labour
*virtually certain*	>99%	<2.6	<2.6	>0.31	>0.28
*extremely likely*	>95%	<3.7	<4.7	>0.29	>0.27
*very likely*	>90%	<4.1	<5.0	>0.27	>0.22
*likely*	>66%	<4.9	<6.2	>0.19	>0.15
*unlikely*	<33%	>6.0	>8.0	<0.14	<0.12

### Distributed model outcomes

The period of smelting activities on Elba started in our model after 360 bce and ended before 139 ce (Table 2). The modelled point of the strongest increase in felling dates between 146 bce and 98 bce (67%; between 237 bce and 79 bce at 95% likelihood) with a median at 117 bce±22 yrs. The strongest modelled decrease in felling activity most likely dates between 95 bce and 51 bce (67%; 130 bce and 36 bce with 95% likelihood; median 75 bce±22 yrs). Maximum felling is simulated to be between 125 bce and 64 bce at a 95%-likelihood and between 100 bce and 75 bce at 67%-likelihood (86 bce±13 yrs). Our data show a relatively smooth increase in fuel consumption between 360 bce and 117 bce±22 yrs. In contrast, the decrease in smelting activities around 75 bce±22 yrs is modelled as relatively rapid. The time lag between the strongest increase in felling activities and the point of maximum felling activities is around 30±22 yrs (1–140 yrs at 95%-likelihood), whereas the time lag is only 5.0±7.4 yrs (0–53 yrs at 95%-likelihood) between the maximum and the strongest decrease in felling activities.

The model suggests that a lack of fuel on Elba most likely occurred between 124 and 85 bce with 67% likelihood (between 237 and 73 bce with 95% likelihood). The likelihood that smelting ended in the 2nd century bce or the first half of the 1st century bce is 6.6% each. In 0.7% of the simulations, all woodland area was cleared before 200 bce.

**Fig 6:**
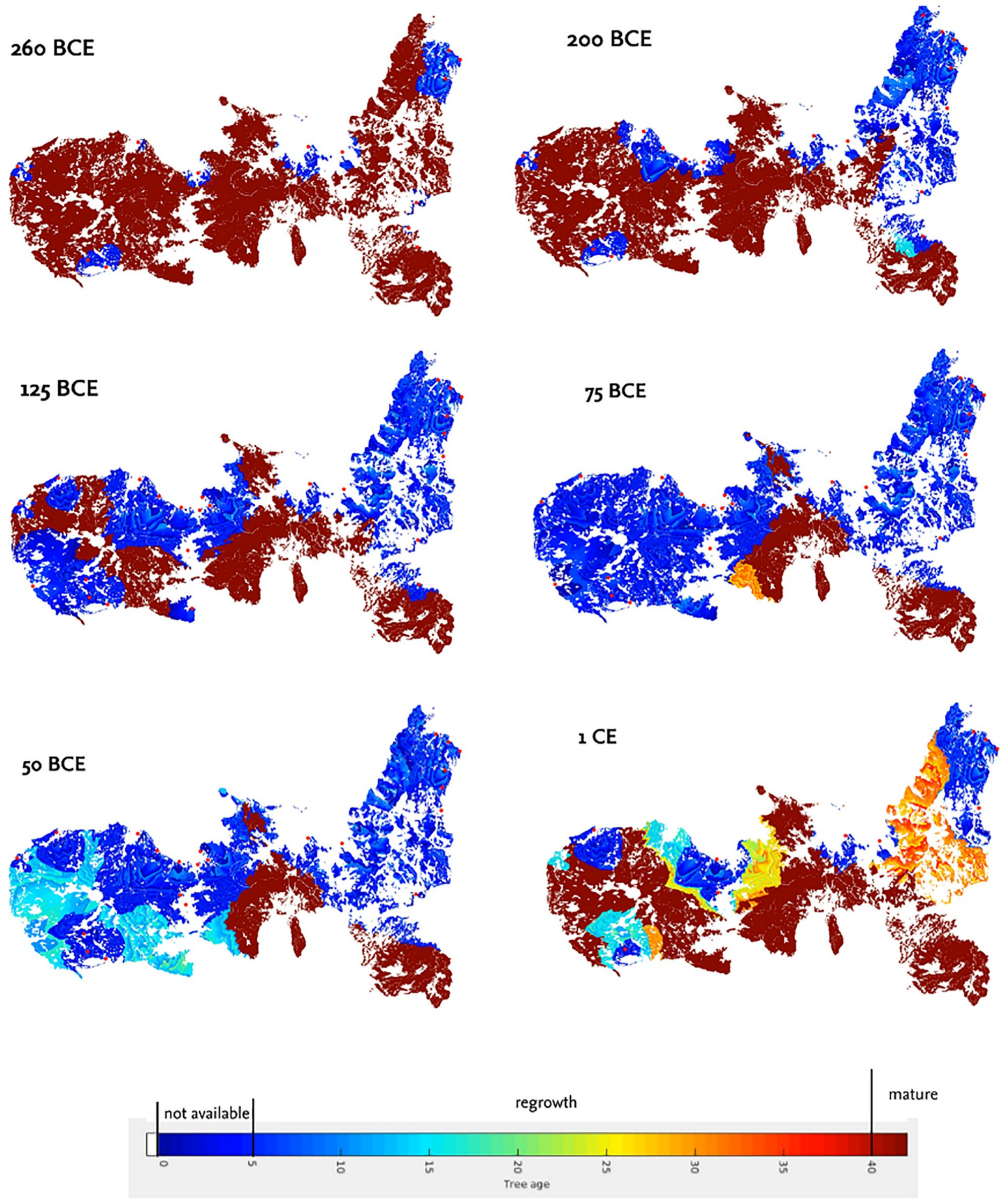
The modelled distribution of woodlot area on Elba in different time slices. Data is from a simulation using the mean values of the parameter distributions as input. Dark blue indicates that trees in the area were not available for harvesting (< 5 yrs)), light blue to orange indicates that the trees in the area are regrowing. Mature trees (> 40 yrs) are indicated in reddish brown.

**Fig 7:**
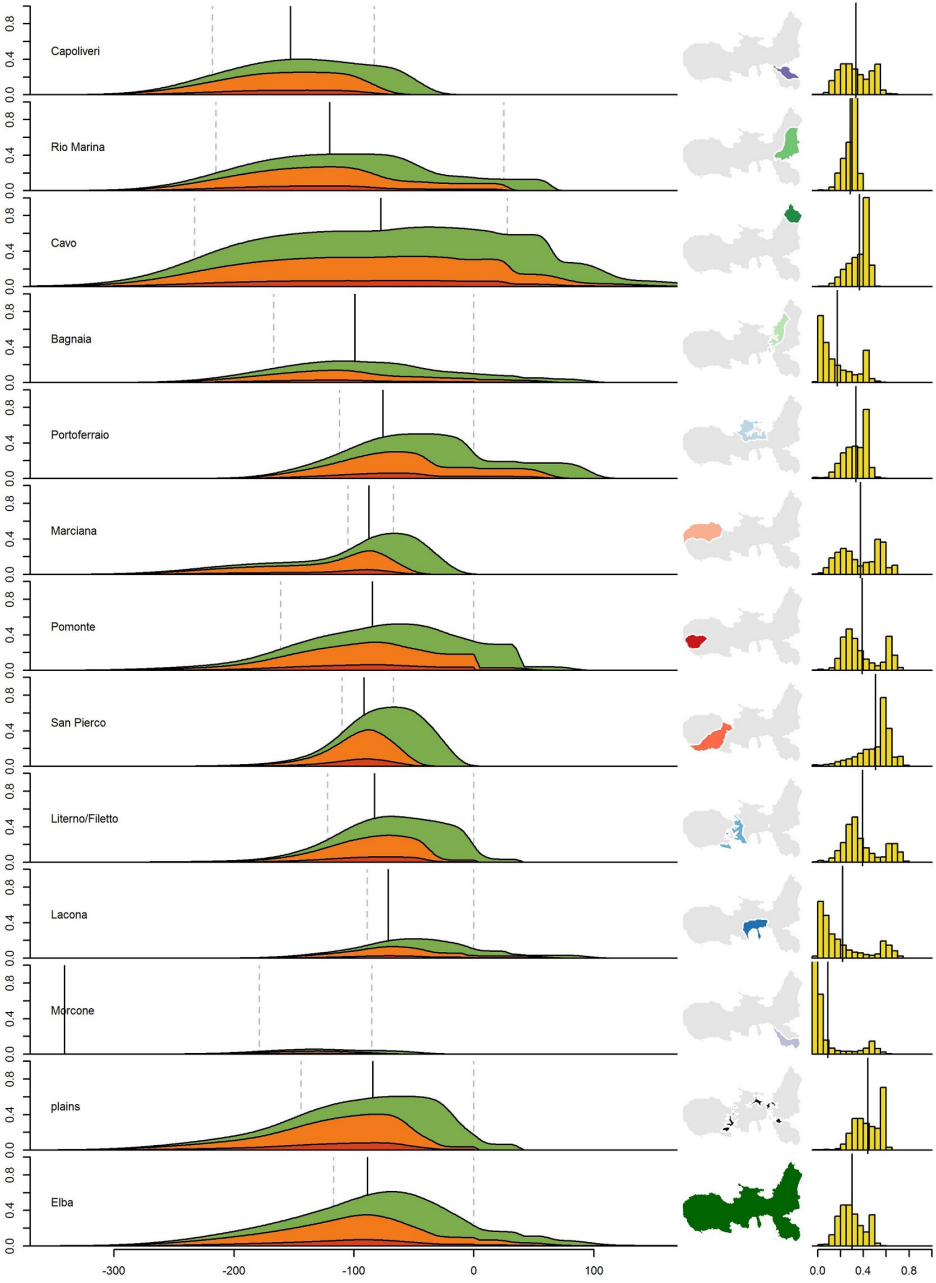
Model outcome for geographical regions on Elba. Order of the regions counterclockwise starting from the northern Calamita-peninsula. **(a)** Average woodlot cover in the regions, red = cleared area, orange = area covered with young woodland not available for cutting (≤5 yrs), and green = woodland available for cutting, but not mature (≤42 yrs, the dashed lines indicate the time of maximal growth/decline in woodland use, the solid line indicates the point in time of maximal woodland use; **b** Location of the regions on Elba; **c** Relative area of the regions covered with woodland not available for cutting (*age* ≤ 5 yrs) in each of the 11,400 simulations, the horizontal line indicates the mean coverage in all simulations.

In addition to the general trend on Elba Island, we also analysed the fuel consumption for twelve geographical regions on Elba separately (Fig 1). The onset and the increase in felling in these regions—as well as the decrease and end—differ clearly (Figs 6 and 7); the earliest onset is simulated for northeastern Elba (Cavo region) and the latest for central Elba (Lacona region). Our data indicate that felling activities first increased in northwestern Elba and the northern Calamita peninsula (Cavo and Rio Marina regions; Capoliveri region). The increase then followed in western and central Elba (Fig 7). There is a general trend of later increase in felling activities from east to west and central Elba. Felling around Pomonte in the far west is modelled to have increased relatively early compared to the general trend, whereas felling around Lacona in south-central Elba started relatively late. The point in time of maximum felling activities around Capoliveri, Cavo and Rio Marina (median between 201 bce and 94 bce) dates to an early period. Maximum felling in western and central Elba is modelled to have taken place later than in eastern Elba (median between 86 and 66 bce; see Fig 7). The latest potential activities are recorded for northeastern Elba (Cavo and Rio Marina regions, mid-1st century bce, Fig 7), whereas in the other regions, felling decreases in the very late 1st century bce. The latest modelled felling activities took place in the northwestern region (Cavo and Rio Marina regions). Felling intensities around Morcone, Lacona and Bagnaia are modelled as low compared to the other regions. For the regions around Morcone and Bagnaia, no ancient smelting sites are recorded. A single, small, smelting site was found in the Lacona-region (Fig 1). The highest average and maximum felling intensities were modelled for the San Piero–Procchio region in western Elba, the region around Cavo in the northwest, and around Marciana and Pomonte in the west (in order of decreasing intensity; see Fig 7).

### Labour

We estimated that during the heydays of iron production on Elba Island, between 1,321 and 3,296 capita were required for the different steps of iron smelting (95%-likelihood; range between 514 and 9,615 capita; 1,842±350 capita, *Mdn*±*MAD*). In increasing order of their relative contribution to the total number of workers, the most important occupational groups were smelters (48.6–57.8% at 95%-likelihood), service staff (37.5%), charcoal burners (3.6–12.1%); and fellers and transport operators (<1% each).

## Discussion

Our results show that the likelihood that no woodland area with wood sufficient for smelting was available on Elba Island is 14% when only the exosomatic metabolism of smelting is taken into account; the likelihood is 27% when both endo- and exosomatic metabolism are taken into account. Therefore, we state that it is *unlikely* that a lack of fuel is the reason for the abandonment—or decline—of smelting on Elba during antiquity. In only 6.2% of our simulations is a lack of fuel simulated for the 1st century bce, which corresponds to the accepted period of the abandonment or decline of smelting on Elba Island (e.g. [33]). None of our simulations resulted in a lack of available woodland on Elba Island in the 1st century ce or later, as production decreased in the 1st century bce.

### Production parameters—The endosomatic metabolism

#### Slag

The modelled number of around 150,000 t of slag that were disposed of on Elba between the 4th century bce and the 2nd century ce is similar to common assumptions on the scale of ancient iron production on Elba [33], which are also based on data from the resmelting concessionaire. Zecchini [32] estimates that between ca. 550,000 and 1,015,000 t slag were disposed of in pre-industrial times (a large amount of it ancient), thus proposing a much greater amount than our figures or the figures of Corretti [33]. However, Zecchini’s figures appear to be rough guesses; figures known from the resmelting concessionaire differ considerably from his figures.

#### Smelting process

The values of furnace efficiency used in our model are in accordance with values reported from experiments [5, 131, 136, 158–160] and other studies on the resource consumption of pre-industrial metallurgy outside the Italian peninsula (Table 5). In smelting experiments conducted on Elba Island, Benvenuti et al. [19] obtained a furnace inefficiency of 2.5 kg/kg and Brambilla [161] a furnace inefficiency of 6.17 kg/kg. Taking the experiment of Benvenuti et al. [19] and an average kiln productivity of.21 kg/kg, our model shows that it would have been *very unlikely* that no woodland area with sufficient wood for smelting was available on Elba during antiquity. When Brambilla’s experiment [161] is taken as a basis, a lack of fuel during antiquity is, however, evaluated to be *as likely as not* (Fig 5). The values of kiln productivity used in our model are relatively low compared to studies conducted for Populonia, but are in the range of other studies on resource consumption (Table 5). Additionally, some studies do not account for the specific gravity of wood (i.e. difference in weight between fresh wood and dry wood used in kilns). Taking parameters used in the other studies, it would be even less likely that iron production on the island suffered from a fuel shortage. However, taking the relatively low kiln productivity used by Goucher [11] or Cleere [3] (or even Saredo Parodi’s exceptionally high furnace inefficiency [10]), complete deforestation of Elba Island in antiquity seems more likely (see Fig 5 and Table 5).

**Table 5:** Main parameters of our model in comparison with other estimates of the resource consumption of early iron smelting. Studies are ordered from low to high. Furnace efficiency in brackets is without preheating and reheating. Notes mark the data source of the respective studies. For studies where a range of values is given, medians were estimated.

Reference	Furnace inefficiency [kg/kg]		Kiln productivity [kg/kg]		Specific gravity [kg/kg]
	median	range	median	range	
Wertime [1]^b^	0.75		.29		–
Voss [12]^b^	0.75		.29		–
Pleiner [4]^b^	[1.40]		.25		–
Healy [8]^b^	[1.5]		.25		?
Brumlich [7]^c^	1.65		.25		.70
Paysen [5]^a^	1.27		.25		.70
Joosten [157]^d^	[1.8]	[1.1–2.5]	?		–
Wallner [9]^b^	[1.9]		.20		?
Williams [53]^b^	1.13		.06		?
Eichhorn *et al*. [14]^b^	3.00		.23	.20–.25	.67
*This study* ^a^	4.2±1.5	1.0–15.6	.21±0.04	.07-.34	.69
Goucher [11]^b^	4.00		.10		?
Cleere [3]^b^	4.00		.14		.60
Saredo Parodi [10]^a^	18.09		.29		–

Notes:

^a^ literature review

^b^ literature

^c^ experimental mass-balance

^d^ chemical mass-balance

Although we principally understand our data as the likelihood of a lack of fuel due to smelting on Elba, the results can also be interpreted with regard to the suitability of the production parameters for iron smelting on Elba. Thus, a simulation run with a given set of parameters that results in a lack of fuel prior to the (presumed) abandonment of smelting sites in the 1st century bce indicates that the applied parameters are unrealistic. This is true for more than half of the simulation runs.

#### Charcoal production

Regarding charcoal production, major issues in the modelling of fuel consumption are the kiln type used and the quality of charcoal required for metallurgical use. As proposed by Veal [112, 140, 162], the ratio of wood to charcoal could even have been higher for the production of high quality charcoal. She assumes that for metallurgical purposes, high quality charcoal is required to achieve higher and constant temperatures. Veal proposes a productivity of .05–.10 kg/kg for high quality charcoal. Taking these values, it is at least *more likely than not* that all woodlands on Elba were cleared for charcoal production. Thus, further knowledge on the quality of charcoal used for smelting is needed. No ancient kilns have been uncovered on the island; consequently, we cannot clearly state if e.g. earth kilns or mound kilns were used during the ancient smelting period. Both mound kilns and pit kilns were known during antiquity, as testified by Theophrastos (*Hist. pl*. 5.9.4; *Hist. pl*. 9.3.1–3; [163–165]). The outcomes of our models are quite different for typical values of pit and mound kilns (0.07–0.17 and 0.13–0.28 kg/kg, respectively [142]); taking values for mound kilns the likelihood that no fuelwood was available on Elba is 13.9%, while for pit kilns it is 34.8%. Given the sheer amount of charcoal necessary for iron smelting on Elba Island during antiquity (*Mdn* = 3.1 Mt, between 1.4 and 7.0 Mt at 95% confidence) and the high required kiln density (3 batches/ha assuming a high kiln volume of 45 t; cf. [76, 166]), some ancient production sites may be found in the future. Several charcoal kiln platforms have been found near medieval smelting sites [48].

#### Site chronology

For our model, we used dating material found on smelting sites to define the operational period of the site; we considered the uncertainty in the chronology by defining certain and uncertain periods of use based on the dating material. Based on the uncertainty in dating some material, our model regonises the end of the smelting period as being later than commonly assumed, i.e. the mid-1st c. bce. However, other finds may allow for the conclusion that smelting continued throughout the 1st century bce. For instance, the Procchio ship wreck (2nd century ce), which carried abundant haematite blocks and sunk close to a known smelting sites [32, 161], documents the use of raw iron ore after the commonly assumed end of the smelting period on Elba Island. Also coin finds from slag accumulations [153], Roman imperial amphorae or so-called *Warzenlampen* [30] indicate smelting activities in the 1st century ce.

Most authors assume that iron smelting on Elba was abandoned (or declined) in the 1st century bce. The simulation runs in our model resulting in a lack of fuel (14% of the simulations taking only exosomatic metabolism into account) date the abandonment between the mid-3rd and the first half of the 1st century bce. Dated material clearly indicates that activities continued at least into the 1st century bce. The likelihood of a lack of fuel in the 1st century bce is 6.2% in our simulations.

### Labour—The endosomatic metabolism

We estimated the impact on wood availability on Elba Island caused by supplying workers involved in ancient iron processing and service (the endosomatic metabolism). The fuel consumption for supply and the impact of browsing goats and grazing sheep/cattle reduces the availability of woodland by between 3 and 100% compared to simulations where only the demand for charging the furnaces is modelled. The likelihood that no woodland with sufficient wood for smelting was available increases from 14% (only exosomatic metabolism modelled) to 28% (exo- and endosomatic metabolism modelled). Thus, the likelihood of there being a lack of fuelwood for smelting during antiquity doubles, although the area required for both exo- and endosomatic metabolism is only 1.19 times higher than the requirements for just the exosomatic metabolism. Therefore,—although according to our simulations it is still *unlikely* that there was a phase where no forest was available on Elba during the ancient smelting period—the contribution of food and fuel supply for the work force is an important factor when calculating the availability of secondary resources for iron smelting.

According to our simulations, around 1850 capita were employed in iron smelting and services in the phase of highest iron production during Roman times (95%-confidence interval between 1,300 and 3,300). This does not include the work force necessary for mining, although in northeastern Elba, a significant amount of iron ore was extracted.

#### Mining

As stated by Corretti [33], figures on the amount of ore extracted on Elba Island are simply wild guesses. The figures provided for ore extraction on Elba Island and iron smelting around the Gulf of Follonica vary widely [1, 10, 12, 128, 167–173] (see Supporting information). It needs to be noted that our rough estimate (see Supporting information) only takes production in the centres around the Gulf of Follonica into account, although it is known that ore from Elba Island was transported to distant locations around the Tyrrhenian Sea [174, 175]. What renders the estimation of persons involved in mining difficult is the fact that we have no data to hand that gives information on the intensity of extraction over the centuries. The intensities given by Saredo Parodi [10] may represent an overall trend, but they remain speculative. With the available data, it seems that the number of workers could have been increased by around 500 capita in the phase of maximum extraction.

The estimated number of workers employed in iron metallurgy on Elba appears to be on an intermediate level compared to numbers estimated for other important *ancient* mining districts (cf. [8, 176–178], and Strabo 3.2.10, Xen. Poroi 4.23 f., Diodor 34.2.19). We are aware that the estimation of the number of workers involved in extraction is difficult. The wide range of estimations of the number of workers employed in the ancient silver mines in Laureion (Attika) illustrates the problem. Here, figures for the 4th century BCE vary by more than one order of magnitude (between 5,000 and 60,000 capita, cf. [168, 179, 180]). However, our rough estimations help to understand the scale of requirements for the workforce and the potential impact on the area required for the supply. We additionally argue that a parameterised estimation of the labour required for all the different steps of iron production on Elba is rewarding to obtain useful figures for the workforce.

### Assumptions

Due to the limited knowledge about various historical conditions on Elba, uncertainty in the estimation of parameters is in part high. Some simplifications are necessary for the model setup as well. Nevertheless, we assume that our model sufficiently covers the main factors of the (fuel) wood consumption on Elba from the 4th century bce to the 2nd century ce.

#### Smelting

For production on Elba and in Populonia, different furnace reconstructions have been proposed [102, 104, 181, 182], but at the moment the record is restricted due to disturbed contexts of the finds (see Section). We therefore assume that during the ancient smelting period, smelting and charcoal burning technologies—and thus furnace inefficiency and kiln productivity—did not change importantly. The data on smelting experiments reported by Nikulka [134] suggest that there is only a relatively small difference between the inefficiencies of different furnace types. However, variability during each furnace batch and between subjects is also reasonable (e.g. experimental data in [134]). Therefore, the outcomes of each Monte Carlo simulation reflect average production parameters during the period of smelting activity.

We assume that the production intensity at each smelting site did not change over time. An overall change in the output of iron production on Elba is nevertheless included in our model. It is assumed that few (small) sites were in operation during an early phase while a large number of (larger) sites operated during an intensive phase (see Table 2). The overall trend of modelled production as shown in Fig 7—an increase in the 2nd century bce and a decrease in the 1st century bce—is commonly found in the literature [33]. The varying intensities of iron production on Elba Island coincide with major political events in the Roman period, such as the Second Punic War, the beginning of Rome’s civil wars, the occupation of new provinces such as *Gallia*, and the transition from the Republic to the Empire.

#### Fuelwood production

In our model, felling preference is a function of transport *cost*. We did not model species selection, although it is known that some *macchia* species were preferably used at the Follonica–Rondelli site [86]. Nevertheless, *e.g*. *Quercus* species were also suitable as fuel [104]. We additionally presume that coppicing was the common practice for fuelwood production on Elba Island, because only small branches of wood are necessary for charcoal production. Coppiced woodlands (*silvae caeduae*, [183]) with various species (e.g. sweet chestnut or oak) are mentioned by several ancient authors (e.g. Theoph. *Hist. pl*. 4.8.11). High *macchia* coppices were also common due to their high calorific value and the suitability for coppicing (presence of a lignotuber, resprouting after disturbances) of some species (especially *Erica* spec. [184–186]). Meiggs [23]states that coppicing was common in silviculture to produce fuel for metallurgy.

We think that it is reasonable to assume that no fuelwood or charcoal was transported from the mainland to Elba Island. The shipping of fuelwood is mentioned e.g. in one ancient text from the 4th century ce, that focuses on the use of wood for heating baths in Rome [23]. The volume of ore required to produce one unit of iron is much lower than the volume of charcoal required to produce the same amount of iron. Therefore, it appears more reasonable that in case of fuel shortage, ore was transported from Elba Island to the mainland than that charcoal (or even fuelwood) was transported to Elba Island. It is archaeologically and historically assured that even in times of sufficient fuelwood supply, ore was transported to the mainland. As some of the smelting sites on Elba Island are located at a great distance from the mines (Fig 1), we assume that the raw ore was transported to these sites by sea. As concluded from interpretations of Diocletian’s *Edict on Maximum Prices* (early 4th century ce), over longer distances, transport by sea is more rational than land-transport [187].

We assume that the woodland cover on Elba Island in antiquity was similar to the present cover building on the assumptions that (1) the woodland was only considerably changed after smelting operations on Elba Island occurred; (2) the present vegetation is relatively dense as regrowth started after the primary sector lost its importance on Elba in the 1950s [149]; and (3) the present land cover (especially on the slopes) is controlled by major ecological factors, mesoclimate (exposition) in particular. North-exposed slopes are more densely covered than south-exposed slopes (see Fig 1). Further modelling approaches on fuelwood consumption could also implement a climate-driven change in vegetation regrowth or the impact of succession or accelerated soil erosion after felling on vegetation regrowth and stand. Degradation of vegetation (or even soil) is not incorporated in our model; only the impact of forest browse is considered as a factor reducing the regrowth of woodland.

Our model has a 1 -yr resolution. Thus, seasonality is not considered in our model, although it might have played an important role in the ancient iron production cycle. From medieval–modern times it is reported that especially the felling of wood is a seasonal task (e.g. [48, 188]).

#### Other fuelwood consumers

Our model mainly takes the wood consumption for smelting operations and the supply of workers employed in the metallurgical production metabolism into account. We did not model the wood demand for fire setting in the mines. We assume that fire setting was not important for mining on Elba Island. In antiquity, mainly near-surface ores were extracted that were relatively easily recoverable. Apart from iron production, no other pyrotechnical industries on Elba (e.g. copper, glass, brick or pottery production) contemporary to the smelting period are archaeologically recorded on Elba. During the middle to late Imperial Age—after the smelting period—, lime-slaking might have taken place on Elba Island [189]. Forging of raw blooms took place here but only on a very small scale [33, 39]; further processing is known from Populonia-Baratti on the mainland [40]. It appears unreasonable that shipbuilding took place on Elba Island, though small scale overhaul work might have been carried out. The activities in the *villae maritimae* and their *partes rusticae* (mid -1st century bce to 1st century ce) might have had significant influence on the land cover. Orcharding is known from Le Grotte [190]. Agricultural production or fuelwood consumption for baths might also have been important in the context of the main city of *Fabricia* (Portoferraio). Scholars considered bathes as important consumers of fuel [113]. Nevertheless, documented food production and bathing post-date the phase of intensive iron production on Elba Island.

### Elba as a case study

The question of whether the decline of the smelting economy on Elba Island was triggered by ecological factors is important for understanding Elba’s history and the metallurgical landscape in *Etruria Mineraria*. Besides, we think that the conditions on Elba Island present a test case for a model with a high uncertainty of input parameters. Several factors facilitate the application of the proposed stochastic model on Elba Island. (i) Given the fact that Elba is an island, surrounded by sea, the boundaries of the model are well defined. Although sea-borne transport appears to be much cheaper than land transport (for longer distances in particular), the transport of raw ore from Elba Island to the mainland might have been more economical than the transport of charcoal fuel from the mainland to Elba Island (the mass of charcoal is higher than the mass of iron ore required for smelting; in addition, the bulk density of charcoal is much lower than the bulk density of iron ore). Hence, fuel production for smelting on Elba Island was presumably local; the assumption of a closed system is reasonable. (ii) Additionally, the amount of slag disposed of on Elba Island during antiquity is well documented. Between the 1910s and 1950s—especially in the interwar period—old slag heaps in Tuscany were removed and the slag was re-used in modern blast furnaces throughout Europe. The exploitation of the slag heaps was documented by concessionaires [104, 128]. (iii) Furthermore, scholars have documented pre-industrial smelting sites since the 18th century (e.g. [153, 191–193]). Especially after the late 1950s, huge parts of the island were surveyed. These surveys provide a comprehensive data set on ancient and medieval smelting sites [32, 38, 42, 48, 94, 104]. Although not all sites are dated, Corretti [48] expects that most undated sites located in the interior of Elba are medieval—few exceptions are known [38]. Most of the sites uncovered with large quantities of slag are Roman. Therefore, we expect that a large portion of the slag ‘produced’ in antiquity is known. (iv) Considering the archaeological documentation of the ancient smelting sites, historical interrelations, and ancient texts, the chronology of iron smelting and phases of production intensity are well known (see Table 2 and S1 Table).

### ‘Deforestation hypothesis’—Revisited

Our results clearly indicate that it is at least *unlikely* that Elba Island was deforested during the ancient smelting period. With the set of parameters at hand, it appears possible that iron smelting on Elba could have been conducted without a lack of fuel. It appears worth revising the ‘deforestation hypothesis’ that suggests that the smelting sites on Elba Island were abandoned in the 1st century bce due to a lack of fuel.

Several scholars who argue for the importance of a lack of fuel cite Strabo’s observation that iron was not processed on Elba, but only on the mainland. Strabo does not, however, provide any reasons why ore was not smelted on Elba Island at the time of his observations [43, 194]. In contrast, the relationship between human agency and deforestation (or forest use) was observed by Strabo on the Apennine Peninsula [84, 85, 195]. Moreover, the transport of raw ore from the Elban mines to the metallurgical centre in Populonia-Baratti was part of the production system as early as the 6th century bce, long before iron was smelted on Elba (cf. [194]). Additionally, Strabo’s note that ‘[the iron] cannot be brought into complete coalescence by heating in the furnaces on the island’ may not only be interpreted as indicating the absence of smelting on the island. It may also indicate that the blooms from Elba were impure—as is typical for bloomery smelting—and had to be refined in the forges in Populonia. Sometimes, Strabo’s text is also translated with ‘cannot reduce it into bars in the furnaces on the island’—see [194] or [196] for comments on the interpretation of Strabo’s text. The near absence of evidence for smithing on Elba Island, but remains of large forges in Populonia [40], support this interpretation. Second, information from (limited) pollen data indicates a shift in the species composition—from deciduous oak to woody shrubland species *(Ericaceae)*—rather than a loss of woodland cover. *Erica arborea* and *Arbutus* were common fuelwood species in Etruscan and Roman metallurgy [61, 86]. Sedimentological analysis of alluvial sediments have shown that morphodynamics—i.e. the deposition of high-magnitude flood layers, slope deposits, and gravel—in the Campo plain (central Elba) increased during the Roman smelting period. Nevertheless, there is no distinct evidence of island-wide clear-cutting [61]. Reasons other than fuelwood scarcity—such as upheaval in the Roman world between the Republic and the Empire—might have fostered the abandonment of smelting sites. In the 1st century bce, the Roman Republic conquered new provinces with important iron ore deposits. A senate’s decree *(senatus consultum)* (Plin. HN 3.138, 33.78) to save the resources in *Italia* is contemporary to the decline in iron production on Elba Island. The decree emphasises a general trend of resource conservation (either to prevent or cope with scarcities or to focus on resource exploration in the provinces). Last, smelting activities on the island also continued in the 1st or even 2nd century ce, albeit on a smaller scale.

**Fig 8:**
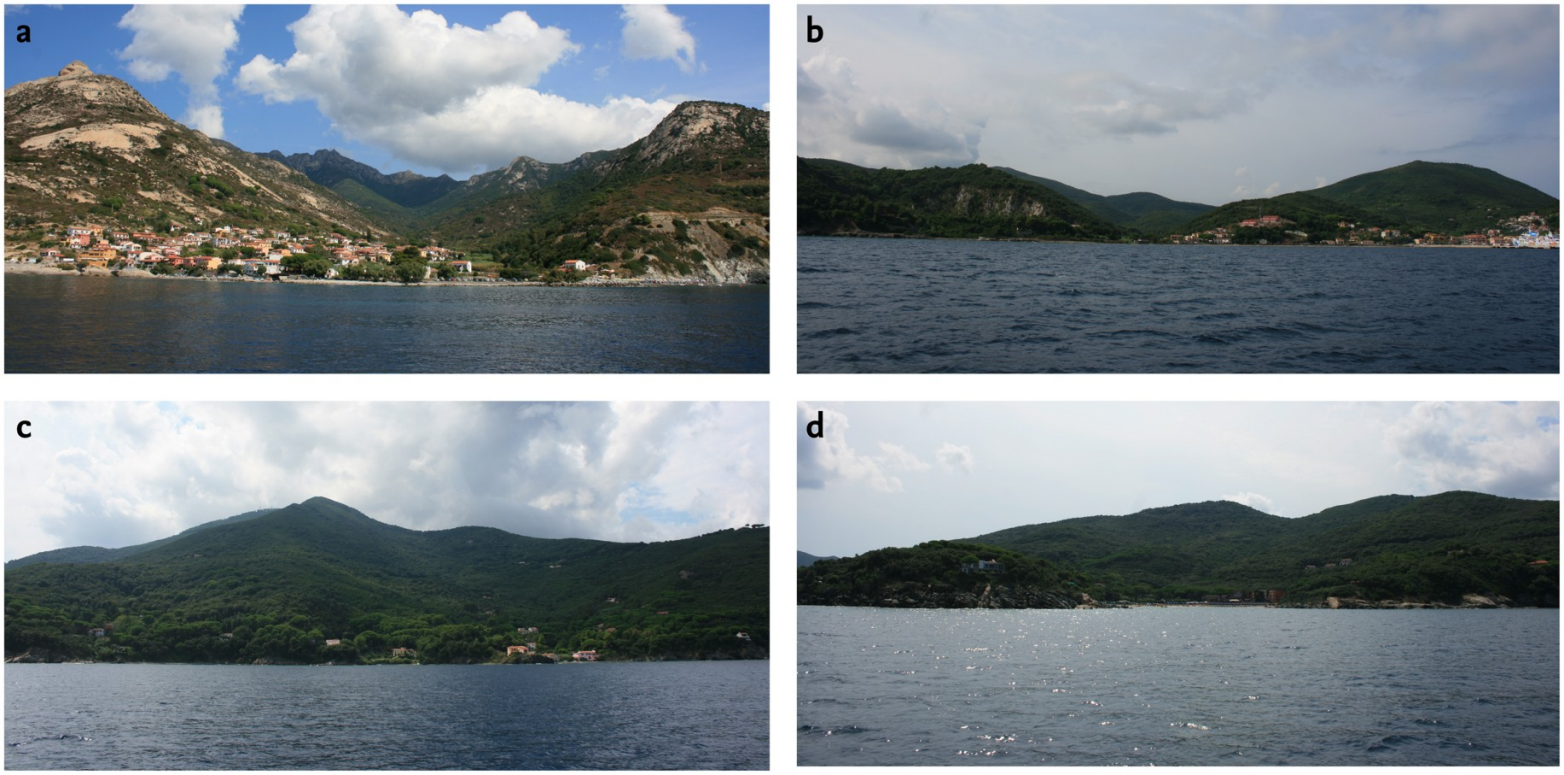
Impressions of valleys on Elba Island where important ancient iron smelting sites were located. (**a**) Pomonte, west Elba; (**b**) San Bennato, northeast Elba; (**c**) Procchio, central Elba; (**d**) Isola della Paolina, northwestern Elba.

The ‘deforestation narrative’ may have developed when Strabo’s text was read in the light of a contemporary impression on the landscape from the 18th to the mid-20th century [50]. In 1814, Schweighardt refers to the situation in antiquity and states that ‘even now, due to a lack of firewood, the ore is not processed on Elba, but on the mainland’ [50]. Most recently, Wiman mentions that deforestation was the reason for the shift of iron production from Elba to Populonia and adds that ‘Elba Island today is still heavily marked by erosion and barren hills.’([34], p. 17). This ‘actualistic’ view of the ancient environment on Elba is similar to Grove and Rackham’s ‘ruined landscapes’ theory [194]. Additionally, in the 18th–early 20th century the perspectives on forests changed clearly. The idea of sustainability emerged [197]. The *problem* of deforestation reached the political agenda and measures tackling (actual or feigned) over-exploitation of forestry became an instrument of power (see e.g. [78, 79] for the *Holznotdebatte*, the debate on wood shortage in 18th century Germany, or [198] for the myths of desertification in colonial northern Africa). Historical documents [199] indicate an expansion of agriculture in the 18th century on Elba Island. Also at this time, a lack of fuel for iron works is deplored [200–202]. In the 1950s—when tourism on Elba Island was in its infancy and the local economy mainly relied on the primary sector—forest cover was far less than today. Agriculture use characterised the plains and the adjacent slopes at this time [149, 203, 204]. With the growing importance of tourism, the land cover and the perception of the island changed. Elba Island was marked as a ‘green island’ (A. Natan: *Grüne Insel Elba—Hohe Grantifelsen, weite Kastanienwälder*, Die Zeit, 09/1968). Today, only a few hills in the northeast of Elba are covered with grassland vegetation, while wide areas of the island are covered by forests [205, 206] (see Figs 1 and 8).

## Conclusions

Our simulations of the woodland required for iron smelting on Elba during antiquity indicate that it is unlikely that a lack of fuel occurred in this period; iron production could have been possible without over-exploiting the woodlands. Thus, other reasons have to be taken into account to explain the abandonment of most iron smelting sites on Elba in the 1st century bce, e.g. access to new iron ore deposits in the Roman provinces. The ‘deforestation narrative’ that is commonly cited in the literature at least since the 18th century therefore needs to be reinterpreted. The narrative may build on the perception of the modern landscape on Elba rather than on a sensitive reconstruction of the ancient conditions. However, the scale of the fuelwood requirement on Elba shows that smelting might have importantly contributed to changes in the landscape, as shown for morphodynamics by Becker et al. [61].

In terms of the socio-ecological model of iron production (Fig 2), the current study shows the importance of considering both the natural and the cultural sphere of causation to better understand ancient iron production. Not only the metabolism of iron production itself, but also the program of smelting (e.g. the demand for iron or access to new ore deposits) and the representation of smelting in modern texts are crucial to understanding human–environment interactions on Elba in antiquity.

Besides the evaluation of the ‘deforestation narrative’, our modelling approach has shown that although we have abundant data on the size and chronology of the smelting sites on Elba, uncertainties are high. An estimation of the likelihood of a lack of fuel is nevertheless possible, especially because of the insularity of Elba. Our model outcomes clearly show the need to take both the endosomatic and the exosomatic metabolism of iron smelting into account when discussing the woodland required for production. Although still unlikely, the likelihood that no fuel was available on Elba for iron smelting in the 1st century bce increased when taking the entire metabolism of iron production into account.

Referring to Theodore Wertime’s dichotomy ‘The furnace versus the goat’ [1], we propose that, based on our model results, both furnaces *and* goats (in the sense of food production) importantly contributed to the availability of woodlot on Elba.

## Supporting information

S1 Appendix Description of model parameters. Description of the procedure to estimate the parameters used for the model, viz. furnace inefficiency; kiln productivity; ore extraction; labour demand: felling, charcoal burning, and smelting, transport, supervision, and service; required farmland, woodland, and rangeland area.
(PDF)

S1 Table Dating material. Overview of dating material used to define the chronology of the smelting sites. Data from literature review, own finds, ^14^C-ages and archive material.
(PDF)

S1 File Computer code of the model. Matlab code of the main settings of the model and the outcomes of an average simulation.
(ZIP)
