# Supplementary material for: The furnace and the goat—A spatio-temporal model of the fuelwood requirement for iron metallurgy on Elba Island, 4th century BCE to 2nd century ce
Source: PLoS One. 2020 Nov 12;15(11):e0241133. doi: 10.1371/journal.pone.0241133 (PMC7661058; doi:10.1371/journal.pone.0241133)
Supplement: S1 Appendix — Description of the procedure to estimate the parameters used for the model, viz. furnace inefficiency; kiln productivity; ore extraction; labour demand: felling, charcoal burning, and smelting, transport, supervision, and service; required farmland, woodland, and rangeland area. (PDF) [file pone.0241133.s001.pdf]

Supplement to: The Furnace *and* the Goat—A spatio-temporal model of the fuel wood requirement for iron metallurgy on Elba Island, 360 BCE to 100 CE

Becker et al.

S1 Appendix: Description of model parameter.

Furnace innefficiency and Kiln productivity as well as site specific parameters are described in the main text.

The labour demand

All labour calculations are based on a 8-hours day and a 300-day year.

Ore extraction

– We estimated the labour requirement for iron mining based on the following parameter:
  - total quantity of slag found on Elba Island, Populonia, and Poggio Butelli
  - chronology of the ancient mining period
  - the quantity of ore that could be extracted by one worker per unit of time
  - a conversion factor from ore to slag from experimental data [1]
– Quantity of slag
  - Scholars estimate that between 2 and 4 Mt of slag were disposed at Baratti beach (Populonia) [2–4]. Estimations from 1920 state that—after small amounts were removed for re-smelting—between 0.4 and 0.6 Mt slag remain in Baratti [5]. From reports of re-smelting contractors, it is known that at least 405,000 t slag were removed in 1921–1932, 1940–1942 and 1960 [5]; this number is in agreement with Crew's assertion [6] that the common numbers are by one order of magnitude overestimated.
  - For Poggio Butelli it is reported that between 1937 and 1939, 149.000 t of slag were re-smelted [5]. Other authors assume that around 0.5 Mt slag were disposed in the area of Poggio Butelli [7].
  - for data on Elba Island see main text
  - Taking the data from re-smelting contractors as a baseline and assuming that the estimations given above are relatively high, we estimate that between 0.71 and 2.7 Mt slag were disposed.
– Slag-to-ore
  - average ore-to-slag ratio from experimental data (1.35 kg/kg [1])
  - 0.96 and 3.65 Mt ore were at least extracted on Elba Island in antiquity;
  - the most important iron ore deposits in ancient *Etruria Mineraria* (i.e modern Tuscany and northern Latium) were located on Elba Island
  - Sperl (cit. in [4]) estimated that 1.5 Mt ore were processed in Populonia.
  - As for some sites (e.g. Follonica–Rondelli) no data on the amount of slag recovered is available, the estimations can only give a lower basis; ore shipped from Elba Island to remote areas is not included in the calculation.

  - The scale of ore extraction assumed by Haupt [8,9] in the late 19th c.(11 Mt ore extracted in Rio since the Iron Age) only fits to our estimation if assuming a large scale medieval extraction.
– Chronology and intensity
  - between 600 BCE and 200 CE the intensity of production changed
  - As most of the archaeological record in the mines got lost during later mining operations, it is only possible to give a very rough estimate of the extraction intensities during the centuries
  - Estimations of the annual extraction on Elba Island during the ancient mining period reach up to 10000 t/yr (around 200 BCE [10])
  - we based our estimation on the values given by Saredo Parodi [4] and the assumption that extraction strongly decreased after the mid-1st c. BCE [11]
– Per-capita-extraction
  - From the annual extraction in each year x_i we calculated the number of workers necessary for the mining operations by applying a factor of 23 t/capita/yr, as given by Harrison [12] for ore extraction in Mouros (including ore preparation and transport)
  - Estimations of labour investment for different extraction operations in antiquity range from 3.5 t/capita/yr in the silver mines of Karthago Nova [13] over 30 t/capita/yr for limestone removed during the construction of the Corinth canal in the 19th c. [14,15] to 60–90 t/capita/yr extraction of auriferous material in northwestern Spain during Roman times [16] (see [17]: 27 t/capita/yr).
  - for iron ore mining in ancient Elba Island, a relatively low number seems to be appropriate
  - in modern mining period on the island, when explosives were used to aid mining, a productivity of 93 t/capita/yr can be reconstructed [18]. This value marks a upper bound.

Charcoal burning

– We calculate the number of workers to run one kiln-batch in a given time for a) pit kilns and b) earth mound kilns.
– For pit kilns we assume an average wood load of 3.5 t/batch and 12.5 man-days/batch for operation, which includes the excavation of the pit, the observation of the burning process, and the discharge of charcoal [19].
– Average mound kilns in our model have a wood capacity of 45 t/batch and a work load of 25 man-days/batch. For a large pit kiln, FAO calculated a work load of 22.5 man-days [19]; Saredo-Parodi [4] assumes a production of 0.7 t of charcoal (so ca. 3.5 t wood, see above) with the afford of 14 man-days (presumably in a pit kiln); FAO [19] reports for a 69 t earth mound kiln a labour investment of 21 man-days.

Felling

– The labour force necessary for the felling of trees is calculated from the modeled woodlot area as described above.
– We assume that the productivity of woodcutters is 13 ha/capita/yr.
– In a trial clearance of oak trees with flint axes <35 cm in diameter found that 500 m^2 of a forest could be cleared by 3 persons in 4 hours [20] (i.e. 8.6 ha/capita/year); they additionally report that in Canada in the 18th c., 17.1 ha/capita/year could be cleared using steel axes.
– During Etruscan/Roman times, workload might have been less than the modern values, but felling tools might have been more efficient than the flint axes; our value approximately equals the average of the two values reported in literature.

Smelting

- According to Saredo-Parodi [4], a 18 kg bloom could be produced per batch in 2 days by 3 smelter.
- As labour investment for smelting is calculated based on the amount of slag found on a site, we used a bloom-to-slag ratio of 0.35 kg/kg to get slag output per furnace batch.
- Accordingly, the average productivity of a smelter in our model is 2.5 t/capita/year (slag).

Land transport

- The calculation of the transport capacity of charcoal on Elba Island is based on the transport capacity of mules and donkeys as reported by Mitchell [21] (mule: 150–180 kg load, 20–24 km/d; donkey: 80–100 kg load, 24–30 km/d).
- We assume an average distance of 2 km between kiln sites and smelting sites.
- Thus, a transport animal carrying 100 kg/capita over 25 km/day on a 4 km track (including return) has a capacity of 187.5 t/capita/yr.
- We used the amount of charcoal necessary for each site to calculate the demand on transport animals on Elba Island.
- Approximately 1 driver might have been necessary to guide 5 animals.

Sea transport

- Smelting sites are located in different locations than the mines on Elba Island.
- As outlined by e.g. Nihlén [22], raw ore was mainly transported by ship around Elba Island.
- For our mode, we ignore the transport of ore from a beaching site to the smelting site, as most sites are located directly on the coast; only relatively some small sites (La Pila and Forcioni) are located in the inland.
- we assume that it took 2 days to travel from the mining area to a smelting site by ship in average
- typical ship had a load of ca. 45–65 t [4, 23, 24]
- we estimate that a ship had a crew of 6–8 persons [25, 26]
- the same number of persons would have been required for (un)loading the ship [4])
- we determined a transport capacity of 1 t/capita/day (ore) for our model.

Supervision and services

- We used the figures given by Saredo Parodi [4] to account for supervision (+ 30%) and service (+30%).
- Numbers in the same scale are reported for the supervision of ancient Amethyst mines in Wadi el-Hudi (early 2nd millenium BCE), were 1050 worker, 98 administrators, and 362 guards (ca. 24%) were employed [27].

Farmland

- We used the data and the spreadsheet provided Hughes et al. [28] to calculate the area requirement for the settlement and food supply of ancient people on Elba Island
- some of the parameters we adjusted to local conditions
- The diet of the local population is adjusted based on archaeozoological data from the hilltop settlement Castiglione di San Martino and Populonia [29, 30]

– Human population dynamics are adjusted to the conditions of mainly industrial area; we did not model age or gender specific caloric requirements as originally proposed by Hughes et al. for a typical village. Caloric requirements were set to
  - 3634 kcal/d for worker with heavy tasks (e.g. mining),
  - 3000 kcal/d for medium task, and
  - 2425 kcal/d for light tasks.
– We obtained productivity values of pastures on Elba Island from the FAO-GAEZ data portal.
– Deviating from Hughes et al., we did not model the requirements for ceramic or bronze production or additional wool sheep keeping because this activities might be insignificant on Elba Island during the smelting period.
– The average requirement for agriculture and horticulture on Elba Island based on the given calculation is 2 ha/capita.

Rangeland

– (modified) calculations given by [28] (see also above)
  - estimated area requirement for transport animals is 0.8–1.1 ha/animal
  - he pasture required for meat supply and dairy production is 0.72 ha/capita
  - area required for forest browse is 0.69 ha/capita
– The contribution of fodder from pastures to the diet of the animals varies between 15% (pigs) and 55/60% (sheep/cattle)
– Forest browse is mainly relevant for goats (55%).
– The area required for additional fodder production is included in the total agricultural land area, whereas we assume that feeding with crop residues etc. did not require additional land

Woodland

– We assume that the fuel used in households for heating and cooking was composed of 20% charcoal and 80% firewood [31]
– The fuel consumption of an inhabitant of Elba Island is assumed to be between 0.5 and 2 t/capita/year [31]
– The production of firewood and charcoal to supply the workforce on Elba Island requires additional afford for charcoal burning and felling and thus increases the total number of worker required, which in turn also increases the necessary fuel production. During a test run of our model, it appeared that the additional number of workers necessary to produce the fuel wood for household purposes is insignificant; so, we did not incorporate it in the final model.

References

1. Nikulka F. Frühe Eisenerzverhüttung und ihr experimenteller Nachvollzug: Eine Analyse bisheriger Versuche. In: Fansa M, editor. Experimentelle Archäologie: Symposium in Duisburg, August 1993. Archäologische Mitteilungen aus Nordwestdeutschland Beihefte. Isensee; 1995. p. 255–310.

2. Voss O. The Iron Production in Populonia. In: Sperl G, editor. The First Iron in the Mediterranean. vol. 21 of PACT. Revue du Groupe européen d'études pour les techniques physiques, chimiques et mathématiques appliquées à l'archéologie; 1988. p. 91–100.

3. Wertime TA. The furnace versus the goat: The pyrotechnologic industries and Mediterranean deforestation in antiquity. Journal of Field Archaeology. 1983;10(4):445–452. doi:10.2307/529467.

4. Saredo Parodi N. Populonia: Inferno o paradiso? Il polo siderurgico di Populonia nell'antichità, un tentativo di quantificazione. Con CD-ROM. Aracne; 2013.

5. Pistolesi C. Ferro autarchico. L'Uso delle antiche scorie ferrifere di Baratti, Poggio Butelli e dell'Isola d'Elba nella siderurgia del Novecento. Archivinform; 2013.

6. Crew P. The iron and copper slags at Baratti, Populonia, Italy. Historical metallurgy: journal of the Historical Metallurgy Society. 1991;25:109–115.

7. Costagliola P, Benvenuti M, Chiarantini L, Bianchi S, Benedetto FD, Paolieri M, et al. Impact of ancient metal smelting on arsenic pollution in the Pecora River Valley, Southern Tuscany, Italy. Applied Geochemistry. 2008;23(5):1241–1259. doi:10.1016/j.apgeochem.2008.01.005.

8. Haupt T. Der Bergbau der Etrusker, dargestellt nach Erfahrungen, directen geschichtlichen Nachrichten und mittelbaren Folgerungen. Berg- und Hüttenmännische Zeitung. 1888;47.

9. Davies O. Roman Mines in Europe. Clarendon Press; 1935.

10. Buchwald VF. Iron and Steel in Ancient Times. vol. 29 of Historisk-filosofiske skrifter. Kong. Danske Videnskab. Selskab; 2005.

11. Corretti A. The mines on the island of Elba. In: Naso A, editor. Etruscology. De Gruyter; 2017. p. 445–462.

12. Harrison FA. Ancient Mining Activities in Portugal. The Mining Magazine. 1931;45(3):137–145.

13. Kay P. Rome's Economic Revolution. First edition ed. Oxford Studies on the Roman Economy. Oxford University Press; 2014. Available from: http://search.ebscohost.com/login.aspx?direct=true&scope=site&db=nlebk&db=nlabk&AN=706539.

14. Ardaillon [U+FFFD] Les mines du Laurion dans l'antiquité. vol. 77 of Bibliothèque des Écoles françaises d'Athènes et de Rome. Thorin et fils; 1897.

15. Hopper RJ. The Laurion Mines: A Reconsideration. Annual of the British School at Athens. 1968;63:293–326. doi:10.1017/S006824540001443X.

16. Andreau J. Recherches récentes sur les mines à l'époque romaine, 2: Nature de la main d'ø euvre. Histoire des techniques et de la production. Revue numismatique. 1990;32:85–108.

17. Freise F. Geographische Verbreitung und wirtschaftliche Entwicklung des süd- und mitteleuropäischen Bergbaus im Altertum. Zeitschrift für das Berg-, Hütten- und Salinenwesen im preussischen Staat. 1907;55:199–268.

18. Krantz AA. Geognostische Beschreibung der Insel Elba. Archiv für Mineralogie, Geognosie, Bergbau und Hüttenkunde. 1841;15(2):347–424.

19. Food and Agriculture Organization of the United Nations. Simple technologies for charcoal making. No. 41 in FAO forestry paper. Food and Agriculture Organization of the United Nations; 1983.

20. Coles J. Archaeology by Experiment. Hutchinson Univ. Library; 1973.

21. Mitchell P. The Donkey in Human History: An Archaeological Perspective. Oxford University Press; 2018.

22. Nihlén J. The Prehistoric Iron Industries of Elba: manuskirpt i Tekniska Museet, Teknik- och industrihistoriska arkivet F 51; 1960.

23. Zecchini M. Relitti romani dell'isola d'Elba; 1982.

24. McGrail S. The Shipment of Traded Goods and of Ballast in Antiquity. Oxford Journal of Archaeology. 1989;8:353–358.

25. Casson L. Ships and Seamanship in the Ancient World. Princeton U. P; 1986.

26. McGrail S. Boats of the World from the Stone Age to Medieval Times. [ACLS humanities e-book edition] ed. Oxford University Press; 2009. Available from: `http://hdl.handle.net/2027/heb.31079.0001.001`.

27. Shaw I. Exploiting the Desert Frontier: The Logistics and Politics of Ancient Egyptian Mining Expeditions. In: Knapp AB, Pigott VC, Herbert EW, editors. Social Approaches to an Industrial Past. Routledge; 1998. p. 242–258.

28. Hughes RE, Weiberg E, Bonnier A, Finné M, Kaplan JO. Quantifying Land Use in Past Societies from Cultural Practice and Archaeological Data. Land. 2018;7(1):9. doi:10.3390/land7010009.

29. Banducci LM. Foodways and Cultural Identity in Roman Republican Italy; 2013. Available from: `http://hdl.handle.net/2027.42/102484`.

30. Verola ML, Degl'Innocenti M. I reperti faunistici. In: Pancrazzi O, editor. Castiglione di San Martino. Studia erudita. Istituti Editoriali e Poligrafici Internazionali; 2016. p. 232–247.

31. Veal RJ. Wood and Charcoal for Rome: Towards an Understanding of Ancient Regional Fuel Economics. In: de Haas T, Tol G, editors. The Economic Integration of Roman Italy. Mnemosyne. A Journal of Classical Studies Supplements. Brill; 2017. p. 388–406.
