# Supplementary material for: The furnace and the goat—A spatio-temporal model of the fuelwood requirement for iron metallurgy on Elba Island, 4th century BCE to 2nd century ce
Source: PLoS One. 2020 Nov 12;15(11):e0241133. doi: 10.1371/journal.pone.0241133 (PMC7661058; doi:10.1371/journal.pone.0241133)
Supplement: S1 Table — Overview of dating material used to define the chronology of the smelting sites. Data from literature review, own finds, 14C-ages and archive material. (PDF) [file pone.0241133.s002.pdf]

Supplement to: The Furnace *and* the Goat—A spatio-temporal model of the fuel wood requirement for iron metallurgy on Elba Island, 360 BCE to 100 CE

Becker et al.

S1 Table: Dating material

Dating material found on the ancient smelting sites on Elba and the time span of their chronology. IDs correspond to numbers in the main text. Charcoal samples from stratigraphic layers containing slag were dated by the radiocarbon method and calibrated by using Oxcal v4.3.2 [1] and the IntCal13 curve [2]. n = note, f = figure, S = site number; pl = plate; JN = John Nihlén (cf. [3])

ID	Site	Dating material [-BCE/CE]			Reference
		Find	From	To	
1-1	Martella ant.	Greco-Italic amphora Will 1A, [4]:341–6	-360	-280	[5]:32
1-2	Martella ant.	As	-211	-154	[6]:855
2-1	Ombria	Campanian black-glazed ware, [7]:47	-300	-25	
3-1	San Bennato	Dressel 12	-30	70	[8]:238, Fig. 5
3-2	San Bennato	Morel 2255, [7]:154	-150	-100	[8]:238, Fig. 5
3-3	San Bennato	Morel 2955a 1, [7]:238	-190	-140	[8]:238, Fig. 5
3-4	San Bennato	Morel 2973/4, [7]:242	-150	-100	[8]:238, Fig. 5
3-5	San Bennato	Morel 3121a 2, [7]:248	-170	-120	[8]:238, Fig. 5
3-6	San Bennato	Stratigraphy	-240	-180	[9], p. 304
3-7	San Bennato	Terra Sigillata Italica, [10]	-60	60	[9]:30
3-8	San Bennato	Corsican jar	-300	-1	[8]:238, Fig. 5
3-9	San Bennato	Antoninian As	138	139	[6]:842–843
3-10	San Bennato	Claudian Sesterce	54	54	[6]:842
4-1	Fornacelle	Dressel 1 (?)	-140	-10	[6]:855; [11]:87–8
4-2	Fornacelle	Vespasian As	73	73	[6]:855
5-1	Capo Pero	Morel 7221b, [7]:405	-300	-201	[12]:24
5-2	Capo Pero	^{14}C, stratum, 1985±30 BP	-47	74	unpubl.
5-3	Capo Pero	Late Campanian black-glazed ware	-100	-40	[13]:176–7/n7
5-4	Capo Pero	Dressel 2/4, [14]:101	-50	100	[13]:176–7/n7
5-5	Capo Pero	Morel 2252e 1, [7]	-200	-101	[15]:91–2
5-6	Capo Pero	Morel 7521a 1, [7]	-100	-50	[15]:88–9
5-7	Capo Pero	*Warzenlampen*, [16]:86	-100	15	[13]:176–7/n7
5-8	Capo Pero	^{14}C-age, inferior stratum	-200	-200	[17]:212
5-9	Capo Pero	Corsican jar	-300	-1	[18]
6-1	Fegatella	Campanian black-glazed ware, [7]:47	-300	-25	Vanagolli 1974
6-2	Fegatella	Campanian black-glazed ware, [7]:47	-300	-25	Vanagolli 1974
7-1	Valle d. Giove	Campanian black-glazed ware, , [7]:47	-300	-25	[19]:S361
7-2	Valle d. Giove	Dressel 1, [11]:87–8	-140	-10	[19]:S361
8-1	Vigneria	‘Roman ceramics’	-300	-1	[19]:S369

Continued on next page . . .

Table 1 – *... continued from previous page*

ID	Site	Dating material [-BCE/CE]			Reference
		Find	From	To	
9-1	RM Spiazzi	Morel 1222a 1, , [7]:93	-200	-1	[13]:176–7/n6
9-2	RM Spiazzi	Silver denarius L. V. Acisculus (RRC 474/1a)	-45	-45	[15]:83
9-3	RM Spiazzi	*Warzenlampe*, [16]:86	-30	15	[13]:176–177/n6
9-4	RM Spiazzi	Flavian sesterce	71	80	[15]:73
9-5	RM Spiazzi	Several As-coins	-225	-145	[15]:74
9-6	RM Spiazzi	Several As-coins	-114	-82	[15]:74
9-7	RM Spiazzi	Fragment of a lamp, [20]:nr251	1	50	[15]:87–88/f6
10-1	Barbarossa	Dressel 1 C, [21]:f6	-120	-50	[12]:11
10-2	Barbarossa	Vandermersch MGS VI, [22]:81–7	-260	-210	[19]:294/S343
11-1	Naregno	Vandermersch MGS VI, [22]:81–7	-260	-210	[12]:18; [19]:294
12-1	Straccoligno	Lyding Will 1 E, [4]	-200	-120	[12]:17
12-2	Straccoligno	Vandermersch MGS VI, [22]:81–7	-260	-210	[12]:17; [19]:294
13-1	Lacona	Dressel 1 (?), [11]	-140	-10	[23]:69
13-2	Lacona	Dressel 1 (?), [11]	-140	-10	[23]:69
14-1	Galenzana	Dressel 1 B, [21]:f11	-130	-100	[12]:23
15-1	Seccheto	Roman ceramics	-280	-25	[12]21, [19]:S335
16-1	Sughera	Roman ceramics	-280	-25	[19]:S175
17-1	Pomonte	Dressel 1 B, [21]:f10	-130	-100	[12]:21–22
17-2	Pomonte	Dressel 1 B, [21]:f12	-130	-70	[12]:21–22
17-3	Pomonte	Dressel 1 B, [21]:f12	-100	-60	[12]:21–22
17-4	Pomonte	Dressel 1 B, [21]:f13	-100	-60	[12]:21–22
17-5	Pomonte	Dressel 1 C, [11]	-120	-50	[12]:21–22
17-6	Pomonte	Dressel 1 C, [11]	-120	-50	[12]:21–22
18-1	Patresi	Dressel 1, [11]	-140	-10	[12]:22
18-2	Patresi	^{14}C (2170±30 BP, 2nd peak), lower stratum	-260	-120	unpubl.
18-3	Patresi	Haltern 70, [24]	-80	190	RAE, aug. 2015
18-4	Patresi	Campana A, [7]:47	-220	-40	RAE, sep. 2017
18-5	Patresi	Dressel 1, [11]	-140	-10	RAE, sep. 2017
19-1	S. Andrea	Morel 2614, [7]:191	-200	-101	[12]:13
19-2	S. Andrea	Beltran 2B, [25]	50	150	JN's campaign, 1958
19-3	S. Andrea	Dressel 1, [11]	-140	-10	JN's campaign, 1958
19-4	S. Andrea	Dressel 1 C, [21]:f10	-120	-100	JN's campaign, 1958
20-1	Marciana Marina	Dressel 1 A, [21]:f11	-140	-100	[12]:22
20-2	Marciana Marina	Lyding Will 1 B, [4]:345–6	-290	-240	[19]:141/pl.X
20-3	Marciana Marina	Vandermersch MGS VI [22]:81–7	-260	-210	[19]:S141/pl.X
20-4	Marciana Marina	Dressel 1 B, [21]:f15	-60	-10	[19]:S141/pl.XI
20-5	Marciana Marina	Morel 1312, [7]:103–4	-200	-101	[19]:S141/pl.XI
20-6	Marciana Marina	Morel 2570, [7]:186–7	-210	-140	[19]:S141/pl.XI
20-7	Marciana Marina	Dressel 1 B, [11]	-130	-10	[26]:193
20-8	Marciana Marina	Lyding Will 1 E, [4]:353–5	-200	-120	[26]:193
21-1	Bagno	Campanian black-glazed ware, [7]:47	-220	-40	[19]:S007
22-1	Paolina	Morel 2253d 1, [7]:154	-200	-80	[13]:176/n5
22-2	Paolina	Morel 2554b 1, [7]:184	-160	-125	[13]:176/n5
22-3	Paolina	Terra Sigillata Italica, [10]	-60	60	RAE, sep. 2017
22-4	Paolina	Dressel 1 B, [11]	-130	-10	[26]:191
22-5	Paolina	Lyding Will 1 E, [4]:353–5	-200	-120	[26]:191
22-6	Paolina	Marabini Moevs IV, [27]:59–62	-150	-20	[26]:191

Continued on next page ...

Table 1 – *. . . continued from previous page*

ID	Site	Dating material [-BCE/CE]			Reference
		Find	From	To	
22-7	Paolina	Morel 1222a 1, [7]:93	-200	-1	[26]:191
22-8	Paolina	Morel 2255/7a 1, [7]:154	-200	-135	[26]:191
22-9	Paolina	Morel 2286a 1, [7]:162	-185	-115	[26]:191
22-10	Paolina	Morel 7541a/b 1, [7]:413	-100	-1	[26]:191
22-11	Paolina	*Warzenlampen*, [16]:86	-100	15	[26]:191
23-1	Gnacchera	Campana Ware [7]:47	-300	-25	[19]:S209
24-1	Guardiola	Dressel 1, [11]	-140	-10	[12]:12
24-2	Guardiola	^{14}C, stratum, 2110±30 BP	-204	-46	unpubl.
24-3	Guardiola	Lyding Will 1 E, [4]:353–5	-200	-120	[26]:191/n359
25-1	Campo all Aia	Dressel 1 B, [21]:f10	-130	-100	[12]:12
25-2	Campo all Aia	Dressel 1 B, [21]:f12	-100	-60	[12]:12
25-3	Campo all Aia	Dressel 1 B [21]:f15	-60	-20	[12]:12
26-1	La Pila	Dressel 1, [11]	-140	-10	[12]:18
27-1	Forcioni	Dressel 1, [11]	-140	-10	[19]:20
27-2	Forcioni	Dressel 2/4, [14]:101	-50	100	[19]:20
27-3	Forcioni	Terra Sigillata Italica, [10]	-60	60	[19]:S020
28-1	S. Giovanni	Dressel 1 B, [21]:f16	-40	-10	[12]:15
28-2	S. Giovanni	Terra Sigillata Italica, C.MA stemp, [28]:1078	15	100	[12]:15
28-3	S. Giovanni	Dressel 1 C, [21]:f10	-120	-100	[29]:191/f10
28-4	S. Giovanni	Lyding Will 1 E, [4]:353–5	-200	-120	[29]:191
28-5	S. Giovanni	Py 4 or 4 A, [19]:85/n262	-550	-350	[29]:191
28-6	S. Giovanni	Morel 2252, [7]:153	-200	-101	RAE, mar. 2017
29-1	Magazzini	Dressel 1, [11]	-140	-10	[12]:15–16
29-2	Magazzini	Dressel 1 B, [21]:f15	-60	-20	[12]:15–16
29-3	Magazzini	Dressel 1, [11]	-140	-10	[29]:187
29-4	Magazzini	TSI, [10]	-60	60	[29]:187
29-5	Magazzini	^{14}C, in-slag charcoal	-260	-165	[30]
29-6	Magazzini	Morel 2640, [7]:197–201	-300	-101	[19]:S138/pl.X

References

1. Bronk Ramsey C. Bayesian analysis of radiocarbon dates. Radiocarbon. 2009;51(1):337–360. doi:10.1017/S0033822200033865.

2. Reimer PJ, Bard E, Bayliss A, Beck JW, Blackwell PG, Bronk Ramsey C, et al. IntCal13 and Marine13 Radiocarbon Age Calibration Curves 0–50,000 Years cal BP. Radiocarbon An International Journal of Cosmogenic Isotope Research. 2013;55(4):1869–1887. doi:10.2458/azu$_{j}s_{r}c$.55.16947.

3. Nihlén J. Fynd av äldre järntillverkning på Elba: Manuskript i Lunds Universitetsbiblioteket Arkiv [John Nihléns efterlämnade papper]; 1958.

4. Will EL. Greco-Italic Amphoras. Hesperia Journal of the American School of Classical Studies at Athens. 1982;51(3):338–356. doi:10.2307/147955.

5. Corretti A. Metallurgia Medievale all'Isola d'Elba. All'Insegna del Giglio; 1991.

6. Sabbadini R. I nomi locali dell'Elba [from Aithalia to Gambale]. Rendiconti Classe di lettere e scienze morali e storiche, Istituto lombardo, Accademia di scienze e lettere. 1919;52:835–858.

7. Morel JP. Céramique campanienne: Les formes. vol. 244 of Bibliothèque des Écoles françaises d'Athènes et de Rome. École Française de Rome; 1981.

8. Corretti A, Firmati M. Metallurgia antica e medievale all'isola d'Elba: vecchi dati e nuove acquisizioni. In: Giardino C, editor. Archeometallurgia : dalla conoscenza alla fruizione : atti del Workshop, 22-25 maggio 2006, Cavallino (LE), Convento dei Dominicani. vol. 8 of Beni archeologici–conoscenza e tecnologie. Edipuglia; 2011. p. 229–241.

9. Firmati M, Principe C, Arrighi S. L'impianto metallurgico tardorepubblicano di San Bennato all'Isola d'Elba: Analisi archeomagnetica. Agōgē Atti della Scuola di Specializzazione in Archeologia dell'Università di Pisa. 2006;3:301–308.

10. Pucci G. Terra Sigillata Italica. In: Italiana IdE, editor. Atlante delle forme ceramiche : 2. Ceramica fine romana nel bacino mediterraneo. (Tardo ellenismo e primo impero). Enciclopedia dell'arte antica classica e orientale. Instituto della Enciclopedia Italiana; 1958–1985. p. 365–406.

11. Peacock DPS, Williams DF. Amphorae and the Roman economy: an introductory guide. Longman archaeology series. Longman; 1986.

12. Corretti A. Indagine preliminare sull'attività di riduzione del ferro in età romana all'isola d'Elba. Geo-archeologia. 1988;1:7–39.

13. Maggiani A. Nuove evidenze archeologiche all'isola d'Elba: I rinvenimenti di età classica e ellenistica. In: Neppi Modona A, Cianferoni GC, Costagli MGM, editors. L'Etruria mineraria; 1981. p. 173–192.

14. Bertoldi T. Guida alle anfore romane di età imperiale: forme, impasti e distribuzione. Editoria e Servizi Archeologi; 2012.

15. Monaco G, Mellini V. Memorie storiche dell'isola dell'Elba: Parte archeologica ed artistica: Trascrizione, Commento, Repertorio Archeologico, Note e Indici. vol. 17 of Pocket Library of Studies in Art. Leo S. Olschki; 1965.

16. Thöne C, Vögele H, Messmer K. Die griechischen und römischen Tonlampen. No. 5. Bd in Katalog der Sammlung antiker Kleinkunst des Archäologischen Instituts der Universität Heidelberg. P. von Zabern; 2004.

17. Camporeale G. Gli Etruschi e le risorse minerarie. Aspetti e problemi. In: Domergue C, editor. Minería y metalurgía en las antiguas civilizaciones mediterráneas y europeas 1. vol. 1. Dirección General de Bellas Artes y Archivos and Ministero de Cultura; 1989. p. 205–212.

18. Vanagoli G. Elenco site Rio Marina – Cavo – Rio nell'Elba. Archivio Storico della ex Soprintendenza Archeologia per la Toscana. 1971;n.1 pos. 9 Livorno 8.

19. Pagliantini L. Aithale. L'Isola d'Elba: paesaggi antichi e bacini d'approvvigionamento. Doctoral Dissertation: Dipartimento Di Studi Umanistici, Università di Foggia; 2014.

20. Leibundgut A. Die römischen Lampen in der Schweiz: eine kultur- und handelsgeschichtliche Studie. Handbuch der Schweiz zur Römer- und Merowingerzeit. Francke; 1977.

21. Lamboglia N. Sulla cronologia delle anfore romane di età repubblicana (II - I secolo a. C.). Rivista di studi iguri. 1955;21(3):241–270.

22. Vandermersch C. Vins et amphores de Grande Grèce et de Sicile, 4e-3e s: avant J.-C. Centre Jean Bérard; 1994.

23. Adamoli R, Rigon D. Meloa: Preistoria e storia di una terra elbana. Bandecchi & Vivaldi; 2013.

24. Carreras Monfort C. Carreras Monfort, César: Haltern 70: a review. Journal of Roman Pottery Studies. 2003;10:85–91.

25. Panella C. Le anfore di età imperiale del Mediterraneo occidentale. In: Céramiques hellénistiques et romaines. Les Belles Lettres; 2001. p. 177–275.

26. Zecchini M. Isola d'Elba. Le origini. vol. 65 of Studi e testi dell'Accademia Lucchese di scienze, lettere ed arti. Accademia Lucchese di scienze, lettere ed arti; 2001.

27. Marabini Moevs MT. The Roman thin walled pottery from Cosa (1948 - 1954). In: Memoirs of the American Academy in Rome, 32.1973; 1973. p. 324 S., Taf.

28. Oxé A, Comfort H, Kenrick PM. Corpus vasorum Arretinorum: a catalogue of the signatures, shapes, and chronology of Italian Sigillata. 2nd ed. No. 41 in Antiquitas. R. Habelt; 2000.

29. Corretti A, Benvenuti M, Chiarantini L, Cambi F. The Aithale Project: Men, Earth and Sea in the Tuscan Archipelago (Italy) in Antiquity. Perspectives, aims and first results. In: Cech B, Rehren T, editors. Early Iron in Europe. Editions Monique Mergoil; 2014. p. 181–195.

30. Becker F, Eser RA, Hoelzmann P, Schütt B. The environmental impact of ancient iron mining and smelting on Elba Island, Italy – A geochemical soil survey of the Magazzini site. Journal of Geochemical Exploration. 2019;doi:10.1016/j.gexplo.2019.04.009.
